# Unique pattern of neutrophil migration and function during tumor progression

**DOI:** 10.1038/s41590-018-0229-5

**Published:** 2018-10-15

**Authors:** Sima Patel, Shuyu Fu, Jerome Mastio, George Dominguez, Abhilasha Purohit, Andrew Kossenkov, Cindy Lin, Kevin Alicea-Torres, Mohit Sehgal, Yulia Nefedova, Jie Zhou, Lucia R. Languino, Cynthia Clendenin, Robert H. Vonderheide, Charles Mulligan, Brian Nam, Neil Hockstein, Gregory Masters, Michael Guarino, Zachary Schug, Dario C. Altieri, Dmitry I. Gabrilovich

**Affiliations:** 1 Immunology, Microenvironment, and Metastasis, Wistar Institute, Philadelphia, PA, 19104;; 2 Institute of Human Virology, Zhongshan School of Medicine, Sun Yat-sen University, Guangzhou, China;; 3 Sidney Kimmel Cancer Center, Sidney Kimmel Medical College, Thomas Jefferson University, Philadelphia, PA 19107;; 4 University of Pennsylvania School of Medicine, Philadelphia, PA, 19104,; 5 Helen F Graham Cancer Center at Christiana Care Health System, Wilmington, DE,; 6 Molecular & Cellular Oncogenesis Programs, Wistar Institute, Philadelphia, PA, 19104;

## Abstract

Although neutrophils have been linked to the formation of the pre-metastatic niche, the mechanism of their migration to distant uninvolved tissues has remained elusive. We report that bone marrow neutrophils from mice with early-stage cancers exhibited much more spontaneous migration to tissues. These cells lacked immunosuppressive activity but had elevated rates of oxidative phosphorylation and glycolysis, and much more production of ATP. Their enhanced spontaneous migration was mediated by the binding of ATP to purinergic receptors. In ectopic tumor models and the late stages of cancers, bone marrow neutrophils demonstrated potent immunosuppressive activity. However, these cells had metabolic and migratory activity indistinguishable from that of control neutrophils. A similar pattern of migration was observed in neutrophils and polymorphonuclear myeloid-derived suppressor cells from patients with cancer. These results elucidate the dynamic changes that neutrophils undergo in cancer and demonstrate the mechanism of neutrophils’ contribution to early tumor dissemination.

## Introduction

The role of neutrophils in cancer is controversial, which is the result of seemingly contradictory activity of these cells able to either promote tumor growth or exert antitumor effects^1, 2, 3, 4, 5^. Identification of polymorphonuclear myeloid-derived suppressor cells (PMN-MDSCs), pathologically activated neutrophils accumulating in cancer that are characterized by immune-suppressive and pro-tumorigenic activity, helped to partially resolve this controversy^3^. It is also suggested that some neutrophils that accumulated in cancer and chronic inflammation contribute to tumor development and progression without eliciting immunosuppressive activity^6, 7^. These cells were provisionally termed ‘MDSC-like’ cells^8^. However, the characteristics of these cells and their distinction from control neutrophils in tumor-free hosts have yet to be defined.

Metastasis, or dissemination of tumors to sites distant from the primary tumor, is the leading cause of mortality in cancer^9^. There is strong evidence to support the role of neutrophils and PMN-MDSCs in tumor metastasis^4, 10, 11, 12, 13^. PMN-MDSCs can condition tumor cells at the primary site to facilitate metastasis, possibly through pathways that regulate the production of hepatocyte growth factor and TGF-β to induce tumor epithelial-mesenchymal transition^14^, the production of matrix metalloproteinase 9 to facilitate tumor invasion^15, 16^, direct immunosuppressive activity that promotes metastasis^13^, and by tethering tumor cells to the vascular endothelium to promote lung metastasis^17^. The mechanisms regulating formation of the pre-metastatic niche by neutrophils and PMN-MDSC are much less clear. S100A8 and S100A9 proteins are known to drive the recruitment of PMNs and PMN-MDSC to pre-metastatic sites in colon cancer patients, and PMN, via the production of S100 proteins, can create a positive feedback loop leading to the accumulation of more PMN in the pre-metastatic lung ^18, 19^. However, the mechanism of initial events leading to formation of the feedback loop remained unclear.

A fundamental characteristic of neutrophils is their ability to migrate to sites of inflammation. This process is directed by chemokines, danger-associated molecule pattern molecules, lipid metabolites, and others^20, 21^. However, it is not clear what would drive initial neutrophil migration to an uninvolved, distant site preceding tumor cells in the absence of measurable inflammation. In addition, although recruitment of PMN-MDSC to the tumor site is well-documented^22^, their migration to other uninvolved tissues was not clear. Moreover, it was reported that some PMN-MDSCs have dramatically reduced migratory activity^23^.

Here, we described the two-phase pathological activation of neutrophils in the bone marrow (BM) of tumor-bearing mice and in the blood of cancer patients. The first phase is characterized by an accumulation of MDSC-like neutrophils that lacked immunosuppressive activity but displayed profound up-regulation of glucose metabolism, ATP production and a potent ability to spontaneously migrate. The second phase is characterized by the accumulation of neutrophils with typical features of PMN-MDSCs that, however, were indistinguishable from control neutrophils with regards to their metabolic activity and migratory behavior.

## Results

### Enhanced motility of BM neutrophils in tumor-bearing mice depends on stage of tumor

**Figure 1.**
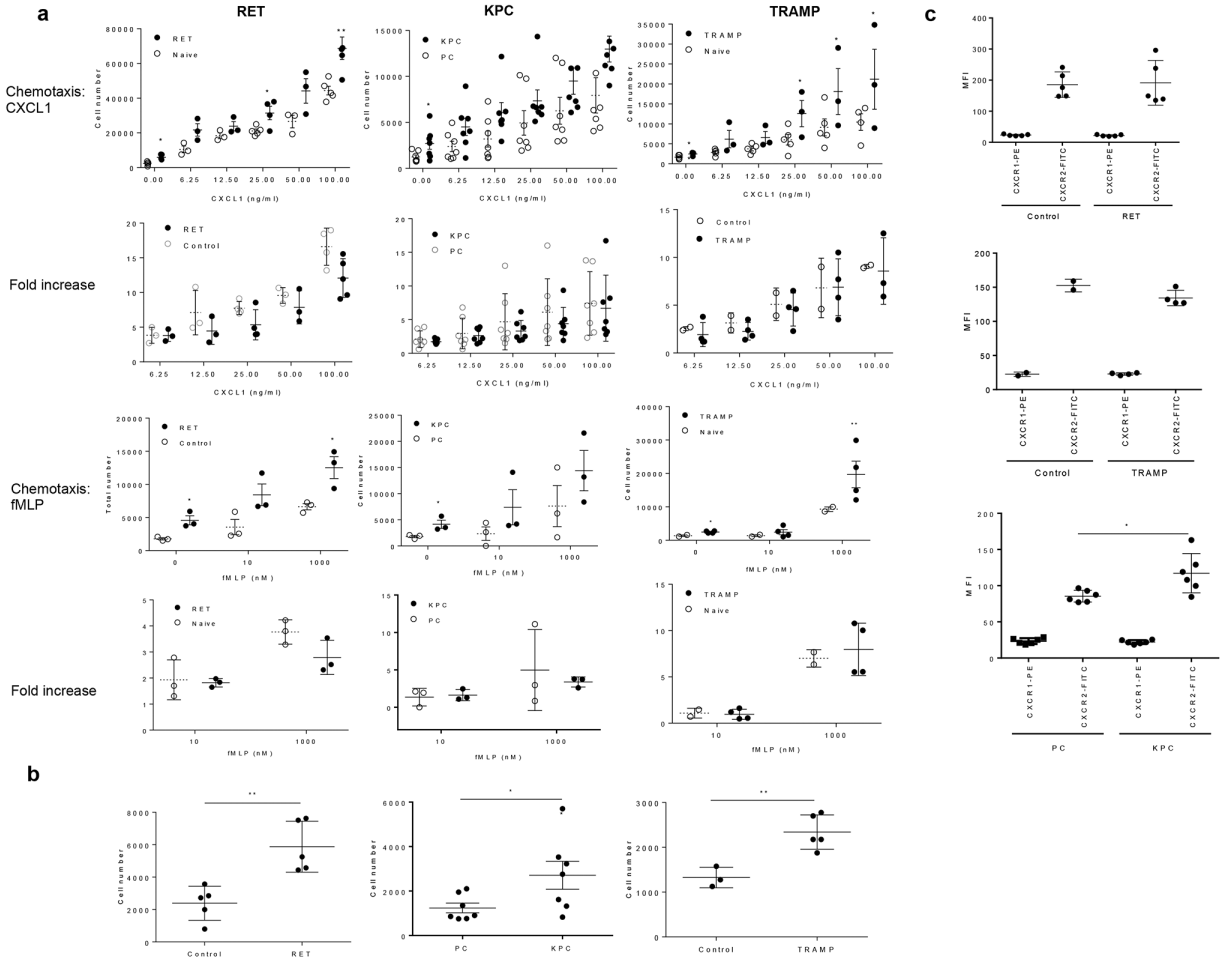
Neutrophils from the bone marrow of three genetically engineered mouse models exhibit increased spontaneous migration. **a**. Transwell assays evaluating the ability of neutrophils to migrate in response to CXCL1 and fMLP stimuli in indicated tumor models. Fold increase is calculated as the ratio of the number of cells that migrate in response to stimulus to the number of cells that spontaneously migrate. For RET model n=3, KPC model n=6, TRAMP model n=3. Mean and SD are shown. P values calculated between groups in two-sided Student’s and are presented as range due to overcrowded figure. * − p<0.05; ** p<0.01. **b**. Spontaneous migration of neutrophils in indicated tumor models. For RET model n=5, for KPC model n=7, for TRAMP model n=5; **c**. CXCR1 and CXCR2 expression on the cell surface of neutrophils. RET (n=5), TRAMP (n=4), KPC (n=6). * − p<0.05; ** p<0.01 for significant differences between control and tumor-bearing mice. Statistics was calculated in two-sided Student’s t-test.

We evaluated the migration of neutrophils isolated from the BM of three different genetically-engineered models (GEM) of cancer: RET melanoma^24^, KPC pancreatic cancer^25^, and TRAMP prostate cancer^26^. These mice were backcrossed for more than 10 generations to the C57BL/6N background. Neutrophils were phenotypically characterized as CD11b^+^Ly6C^lo^Ly6G^+^ and were isolated based on the expression of the Ly6G marker. Migration and chemotaxis of neutrophils were assessed using a standard Transwell membrane assay. In all three models, we observed substantially higher migration of neutrophils from tumor-bearing mice in response to the chemokine CXCL1 or the chemoattractant fMLP than neutrophils from tumor-free littermates (Fig. 1a). However, when the ability of these stimuli to induce cell migration (ratio between stimulated and unstimulated cells) was evaluated, no differences between TB and tumor-free mice were observed (Fig. 1a). Instead, we observed markedly higher spontaneous migration of neutrophils in tumor-bearing than in tumor-free mice (Fig. 1b). No or very small differences in the expression of the CXCL1 receptors, CXCR1 and CXCR2 were evident (Fig. 1c) consistent with the results of neutrophil chemotaxis. Though the total number of neutrophils was significantly elevated in the BM of KPC pancreatic mice, the total number of neutrophils in RET melanoma and TRAMP prostate mice was similar to control mice (Supplementary Fig. 1a), suggesting that changes in motility were not associated with expansion of neutrophils in BM.

**Figure 2.**
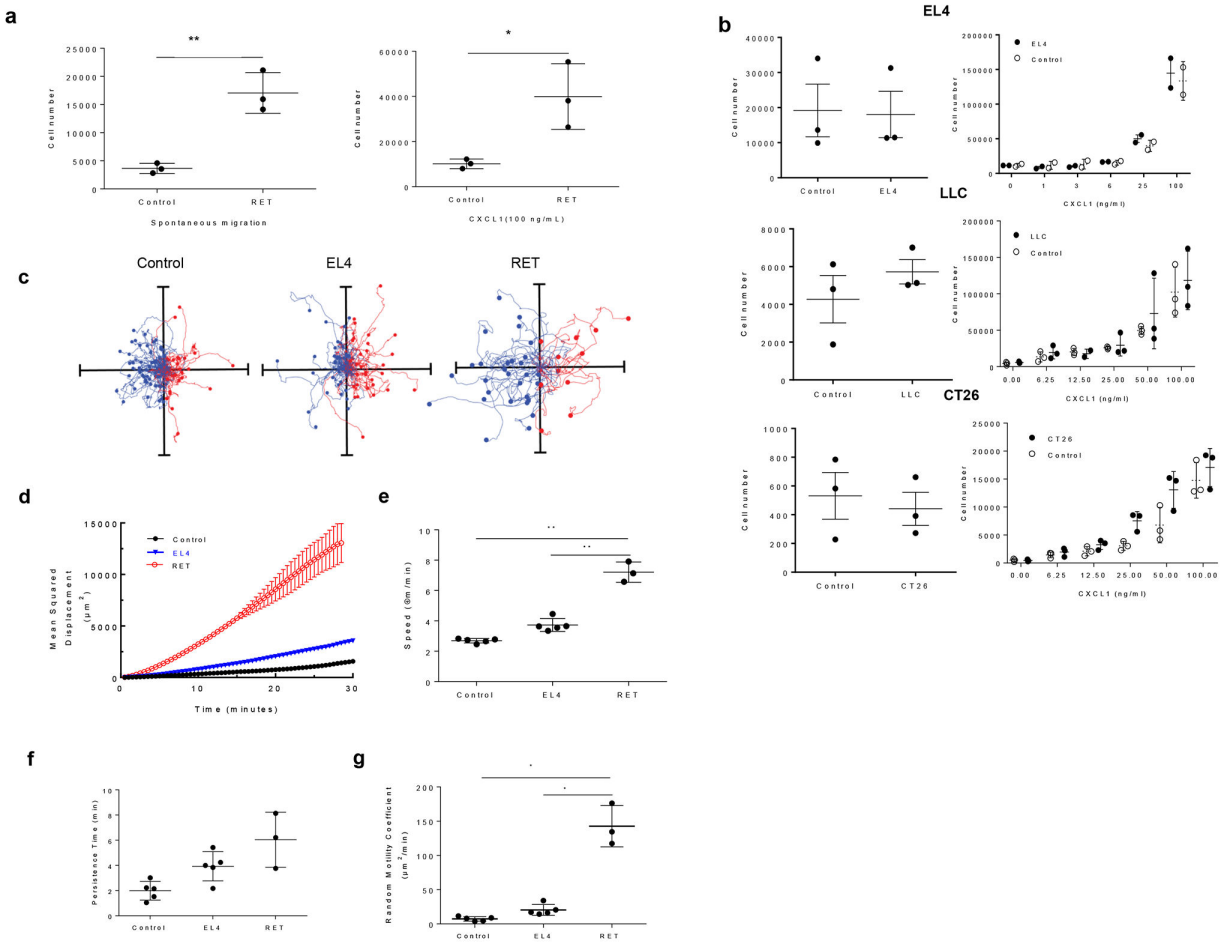
Neutrophils from genetically engineered mouse models, but not transplantable tumor mouse models, exhibit an increased spontaneous migration. **a**. Transwell assays evaluating the ability of peripheral blood neutrophils from RET melanoma mice to spontaneously migrate (left) and chemotax in response to CXCL1 (right). N=3, mean, and SD are shown. **b**. Transwell assays evaluating the ability of neutrophils to spontaneously migrate (left) and chemotax in response to CXCL1 (right). N=3, mean, and SD are shown. **c–g**. Analysis of neutrophil migration with time-lapse video. **c**. Representative cell traces of neutrophils. Axes - 400 μm. **d**. The cell traces and mean-squared displacements of neutrophil migration. **e**. speed, **f**. persistence times, **g**. random motility coefficients of BM neutrophils isolated from control tumor-free (n=5), EL4 (n=5) and RET melanoma (n=3) bearing mice. Mean and SD are shown. * − p<0.05; ** p<0.01 in two-tailed Student’s t-test.

To confirm that differences in migration was not only limited to the BM, we isolated neutrophils from the peripheral blood of control and RET melanoma mice. Neutrophils from RET melanoma mice demonstrated markedly higher spontaneous and CXLC1-induced migration than control neutrophils (Fig. 2a). When we evaluated the migratory behavior of BM neutrophils from transplantable subcutaneous (s.c.) tumor models, EL4 lymphoma, LLC lung carcinoma, and CT26 colon carcinoma, no differences in migration between control and tumor-bearing mice were observed (Fig. 2b). The number of BM neutrophils in EL4 mice was markedly higher than in control mice (Supplementary Fig. 1b). We analyzed cell movement using time lapse microscopy on fibronectin-coated surfaces. BM neutrophils from RET melanoma mice, but not from EL4 mice, demonstrated a higher capacity to spontaneously migrate than control neutrophils (Fig. 2c and Supplemental video), and was characterized by markedly higher mean-squared displacement over time (Fig. 2d), speed (Fig. 2e), persistence time (Fig. 2f) and random motility coefficient (Fig. 2g).

**Figure 3.**
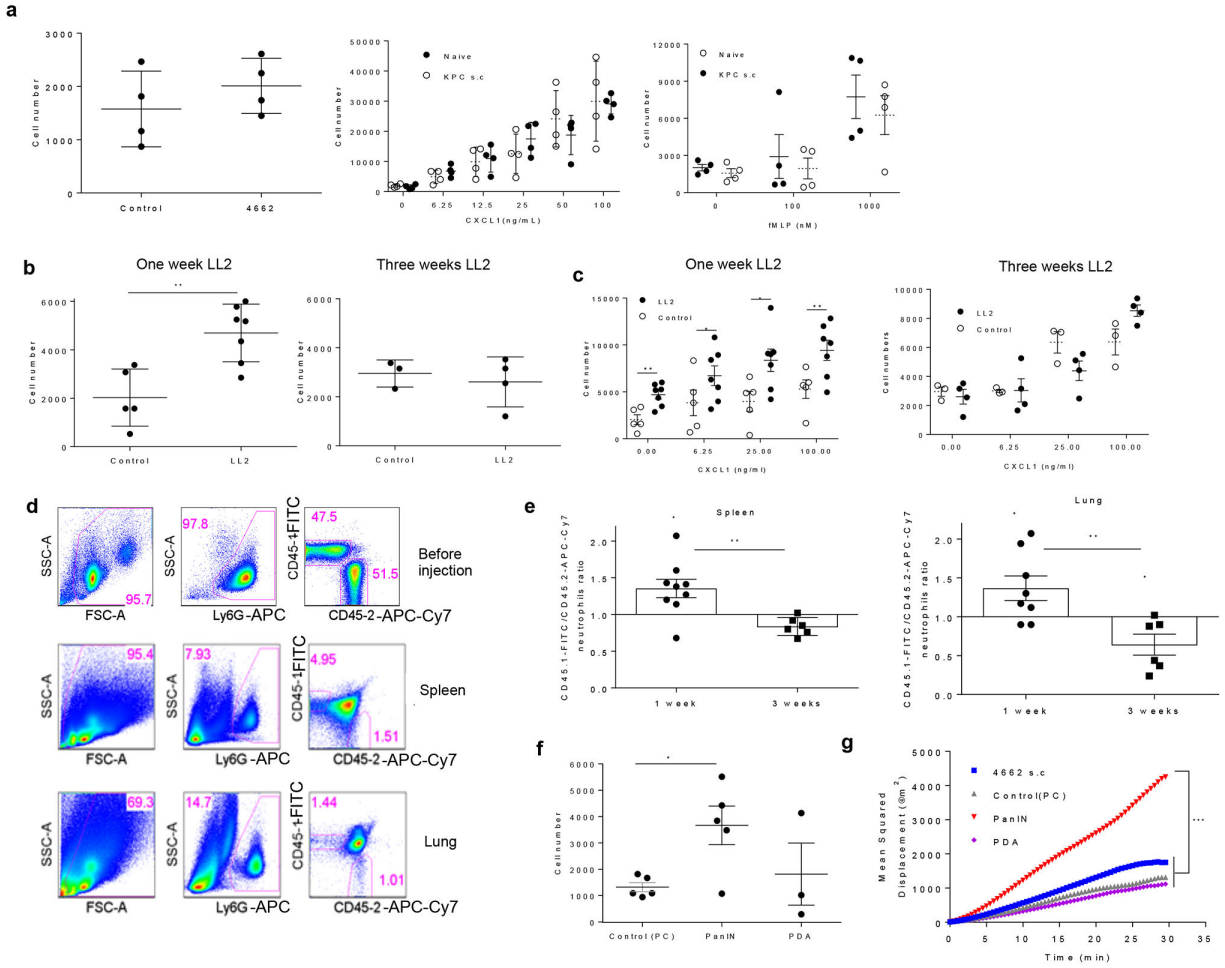
Neutrophils from the early, but not late stages, of an orthotopic lung cancer model exhibit increased spontaneous migration. **a**. Transwell assays evaluating the ability of BM neutrophils from mice bearing s.c. 4662 tumors derived from KPC mice to spontaneously migrate (left) and chemotaxis in response to CXCL1 (middle) or to fMPL (right). Mean, and SD are shown (n=4). P values are calculated in two-sided Student’s t-test (not shown due to lack of significance). **b**. Transwell assays evaluating the ability of neutrophils to spontaneously migrate. In one week LL2 experiments, n=5 for control group and n=6 for LL2 mice. In three week LL2 experiments, n=3 for control mice and n=4 for LL2 mice. Mean, and SD are shown. **c**. Transwell assays of neutrophil chemotaxis in response to CXCL1. Mean and SD are shown. **(d, e)** Flow cytometry analysis of CD45.2^+^ BM neutrophils from control mice and CD45.1^+^ neutrophils from one-week or three-week LL2 TB mice to migrate into the spleen and lung 1 hour after injection to naive CD45.1^+^CD45.2^+^ recipients. **d**. Flow cytometry gating strategy with representative result from 6 performed experiments, **e**. Ratio between CD45.1^+^ and CD45.2^+^ CD11b^+^Ly6G^+^Ly6C^lo^ neutrophils. In each experiment, the ratio of CD45.1^+^/CD45.2^+^ cells injected into recipient mice was set as the baseline= 1. For one week LL2 experiments n=8, for three-week LL2 experiments n=6. Mean, and SD are shown. P values are calculated in two-sided Student’s t-test from baseline within each group of LL2 mice and between the two groups of LL2 mice are shown. **f**. Transwell migration assay of BM neutrophils from control and KPC mice with PanIn and invasive PDA. N=5 (control), n=5 (PanIn), n=3 (PDA). Mean and SD are shown. **g**. The mean-squared displacements of neutrophil migration. Each curve represents cumulative results of traces 45–60 individual cells. In **a–c, e,f** p values were calculated in two-sided Student’s t-test. * − p <0.05, **−p<0.01. In **g**. p values were calculated in two-way ANOVA test with Bonferroni correction for multiple comparisons. ***−p<0.0001 Two experiments with similar results were performed.

To confirm that how the tumor is established in mice has a critical effect on neutrophil motility, we investigated the migratory behavior of BM neutrophils from a transplantable tumor model established by subcutaneously injecting 4662 a subline of tumor cells derived from KPC mice^27^. In contrast to neutrophils from KPC mice, neutrophils from 4662 mice did not display increased spontaneous or chemokine-induced migration (Fig. 3a).

We hypothesized that inflammation caused by injecting a large number of tumor cells at the ectopic site could explain the lack of enhanced spontaneous migration of neutrophils in s.c. transplantable models. To test this hypothesis, we utilized an orthotopic model of lung cancer by injecting a small number (10^5^) of LL2 tumor cells containing luciferase (LL2-Luc) intravenously (i.v.), which resulted in the formation of tumor lesions in the lung within one week of injection and large lesions three weeks after injection (Supplementary Fig. 1c). The total number of BM neutrophils was substantially increased three weeks, but not one week after injection (Supplementary Fig. 1d). BM neutrophils from mice one week after injection had markedly higher spontaneous and CXCL1-induced migration than control BM neutrophils (Fig. 3b, c). However, when cells were isolated three weeks after injection, no differences were observed (Fig. 3b, c). To verify these observations in vivo, we isolated BM neutrophils from congenic LL2 TB (CD45.1^+^) and control tumor-free (CD45.2^+^) mice, mixed them at a 1:1 ratio, and injected them i.v. into CD45.1^+^ × CD45.2^+^ tumor-free recipients. The ratio between CD45.1^+^ and CD45.2^+^ neutrophils was evaluated in spleens and lungs one hour after injection (Fig. 3d). In both tissues, neutrophils from one-week mice were more prevalent than control neutrophils, indicative of increased migratory ability. In contrast, the ratio between neutrophils from three-week and control mice was markedly lower in spleens and lungs (Fig. 3e).

We asked whether association between stage of tumor development and neutrophil activity is observed in mice with GEM of cancer. KPC model could be used to address this question. By 8 to 10 weeks of age, KPC mice develop pancreatic intraepithelial neoplasia (PanIN). KPC mice develop spontaneous pancreatic ductal adenocarcinoma (PDA) with 100% penetrance^25^, and by 16 weeks of age, most KPC mice have developed locally invasive PDA. Therefore, we were able to separately evaluate mice at the relatively early (PanIN) and relatively late (invasive PDA) stage of pancreatic cancer development. We found that only mice with PanIN, but not invasive PDA, had increased spontaneous migration of neutrophils assessed by Transwell assay (Fig. 3f) and mean squared displacement assay (Fig. 3g).

Thus, in GEM of melanoma, pancreatic, and prostate cancer and at early stages of orthotopic lung cancer, BM neutrophils demonstrated a high capacity to spontaneously migrate. In contrast, BM neutrophils from the s.c. transplantable tumor models and the three-week orthotopic lung cancer model (with substantial tumor burden) exhibited migratory activity similar to control neutrophils.

### BM neutrophils in the early and late stages of lung cancer are functionally different

**Figure 4.**
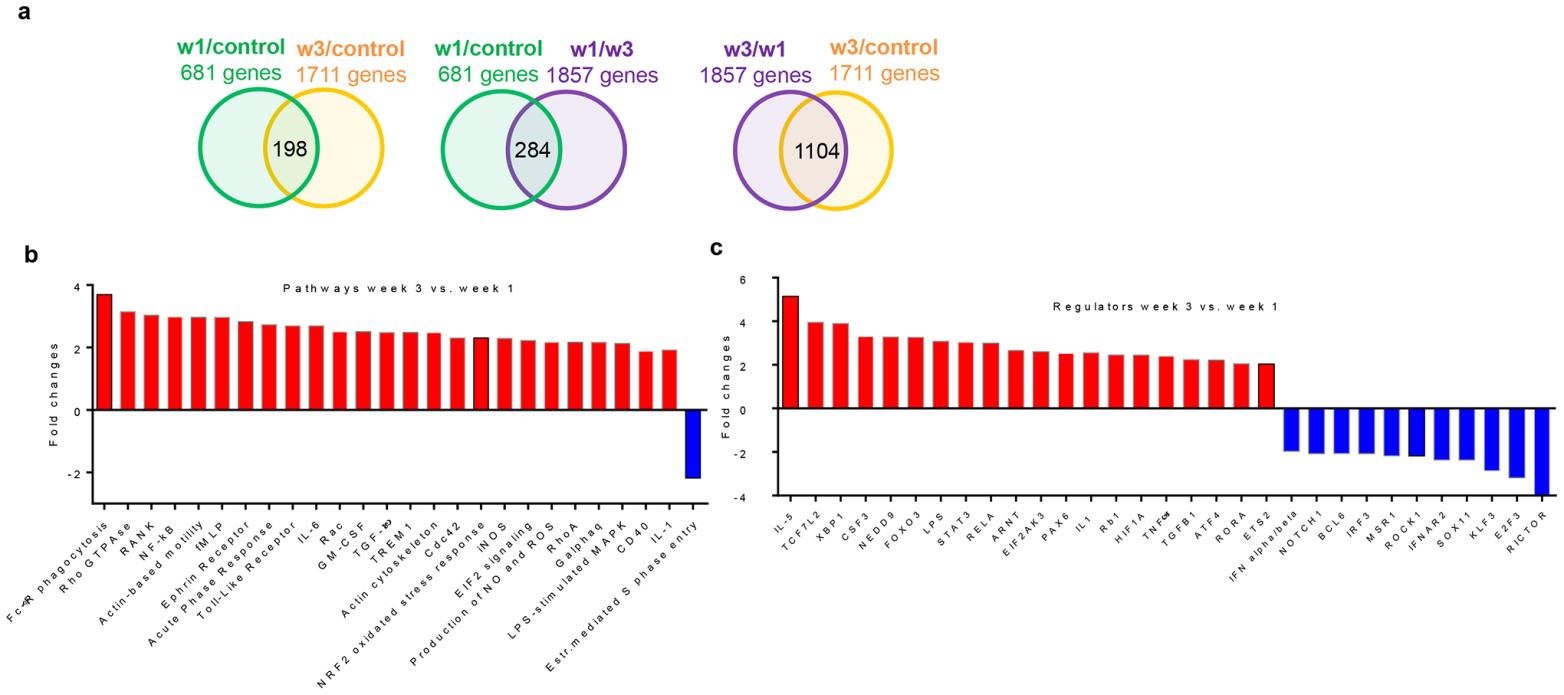
Transcriptome and functional activity of neutrophils in TB mice. BM Neutrophils were isolated from naive, one week and three-week LL2 TB mice. Gene expression profile was evaluated using RNA-seq. Three samples from each group of mice was evaluated **a**. Total number of changed genes, **b**. significant (FDR < 5%) changes in pathways between three-week and one-week TB mice. **c**. Significant (FDR < 5%) changes in regulators between three-week and one-week TB mice.

To investigate the differences between mouse BM neutrophils in early and advanced stages of orthotopic lung cancer, we performed gene expression profiling using RNA-seq. Neutrophils from three-week tumor-bearing mice had more significantly changed genes from control mice than neutrophils from one-week tumor-bearing mice (Fig. 4a). Ingenuity Pathway Analysis (IPA) revealed that the most enriched network in BM neutrophils from one-week, but not three-week, LL2 TB mice was Energy Production, Nucleic Acid Metabolism, and Small Molecule Biochemistry (Supplementary Fig. 2). The EIF2 signaling pathway associated with endoplasmic reticulum (ER) stress was significantly up-regulated in neutrophils from one-week and three-week LL2 mice compared to control neutrophils (Supplementary Fig. 3). Neutrophils from three-week LL2 mice had substantial up-regulation of multiple pathways associated with inflammatory responses, ROS and oxidative stress response, iNOS signaling, and ER stress compared to neutrophils from one-week LL2 mice (Fig. 4b). Most of these pathways have been previously implicated in PMN-MDSC function^28^. Consistent with these results, genes regulated by XBP1, LPS, IL-1, HIF1A, TNF, and ATF4 were increased in three-week LL2 mice over one-week LL2 mice (Fig. 4c). These results suggested that BM neutrophils in three-week but not one-week LL2 mice could be bona fide PMN-MDSCs.

**Figure 5.**
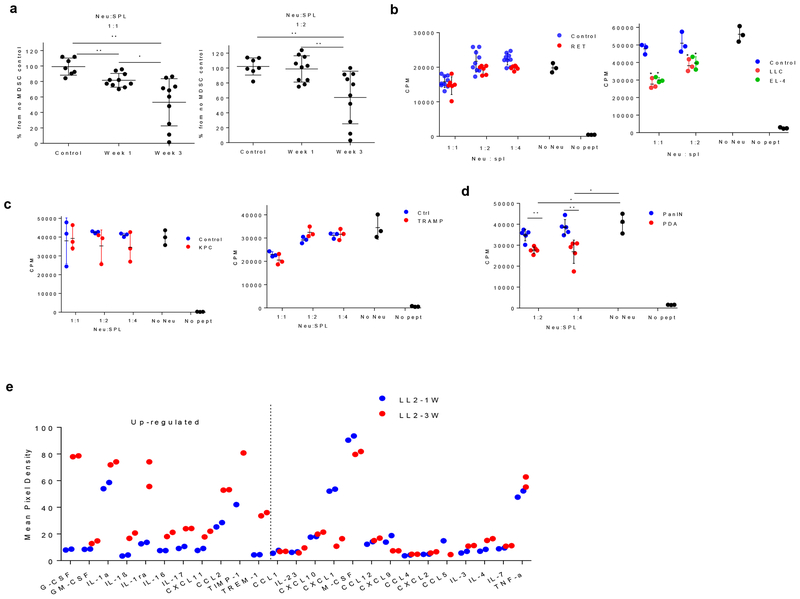
Suppressive activity of BM neutrophils in TB mice. **a**. Antigen-specific proliferation of CD8^+^ T cells in the presence of BM Ly6G^+^ cells isolated from control mice and one or three weeks LL2 mice. Transgenic, Pmel splenocytes stimulated with specific peptide were used as responders. Proliferation was measured in triplicate by ^3^H thymidine uptake. Baseline of T cell proliferation in the absence of added neutrophils was set as 100%. Control -n=7, week 1 LL2 - n=10, week 3 LL2 - n=11. Mean, and SD are shown. **b**. Antigen-specific proliferation of CD8^+^ T cells in the presence of BM Ly6G^+^ cells from naive mice, RET melanoma mice (left panel), LLC, or EL4 (right panel) mice. Pmel splenocytes stimulated with specific peptide were used as responders. Proliferation was measured in triplicate by ^3^H thymidine uptake. One experiment is shown. Three experiments with the same results were performed. Each experiment included 3 mice. Mean and SD are shown. **c**. Suppressive activity of PMN from BM of KPC and TRAMP transgenic mice. OT-1 splenocytes stimulated with specific peptide were used as responders. Proliferation was measured in triplicate by ^3^H thymidine uptake. For KPC mice 5 experiments with similar results were performed. In TRAMP mice two experiments with the same results were performed. Mean and SD are shown. **d**. Suppressive activity of neutrophils from KPC mice with PanIN and PDA. Proliferation was evaluated by ^3^H-thymidine uptake in triplicates. Means and SD of cumulative result from two mice are shown. **e**. Cytokine protein expression array in sera of one- and three-week orthotopic LL2 TB mice. Experiments were performed in duplicates and individual data are shown. On the left cytokines with more than 2SD increase in sera of three-week LL2 mice over one-week LL2 mice. On the right cytokines with less than 2SD increase or decreased in three-week LL2 mice. * − p <0.05; **−p<0.01 in two-sided Student’s t-test.

To functionally verify this observation, we evaluated the ability of BM neutrophils to suppress antigen-specific T cell responses. The neutrophils from one-week tumor-bearing mice showed minimal suppressive activity, whereas neutrophils from three-week tumor-bearing mice exhibited potent suppressive activity (Fig. 5a). BM neutrophils from control mice were not suppressive. Similar to the cells from one-week LL2 mice, BM neutrophils from RET melanoma mice (Fig. 5b), KPC and TRAMP mice (Fig. 5c) did not suppress T cell responses. In contrast, BM neutrophils from s.c. LLC and EL4 mice suppressed T cell proliferation (Fig. 5b). In a separate set of experiments, we compared suppression by BM neutrophils isolated from KPC mice with PanIN and PDA. Neutrophils from mice with PanIN had no suppressive activity. Neutrophils from mice with PDA had substantial suppressive activity (Fig. 5d).

Since ROS production and the oxidized stress response were among the main pathways up-regulated in neutrophils in three-week LL2 TB mice, we evaluated the level of cytoplasmic ROS produced by neutrophils. BM neutrophils from three-week LL2 mice, but not from one-week LL2 mice, had markedly higher cytoplasmic ROS than control neutrophils (Supplementary Fig. 4a). Neutrophils from RET melanoma mice had a ROS production similar to that of control mice (Supplementary Fig. 4b), whereas BM neutrophils from s.c. EL4 and LLC mice had a slightly elevated ROS level (Supplementary Fig. 4c).

To better understand the mechanism regulating functional differences in PMN at early and late stages of cancer we assessed the cytokine profile in sera of mice with one-week and three-week LL2 tumors. We observed up-regulation (more than 2 standard deviations) of number of pro-inflammatory cytokines (G-CSF, GM-CSF, IFN-γ, IL-1β, IL-17, TREM-1, TNF) in mice with three-week tumors as compared to one-week tumor (Fig. 5e).

Thus, based upon these functional characteristics, BM neutrophils from three-week LL2 orthotopic mice, and s.c. LLC and EL4 mice, and, to some extent, KPC mice with invasive PDA were typical PMN-MDSCs. BM neutrophils from one-week LL2 mice and from mice with GEMs of cancer were not PMN-MDSCs. However, they had elevated levels of ER stress response genes. We provisionally called them PMN-MDSC-Like Cells (PM-LCs). This term was coined previously to describe a population of activated neutrophils in cancer that did not acquire full capacity to suppress immune responses^8^.

### PM-LC and PMN-MDSC are metabolically distinct

**Figure 6.**
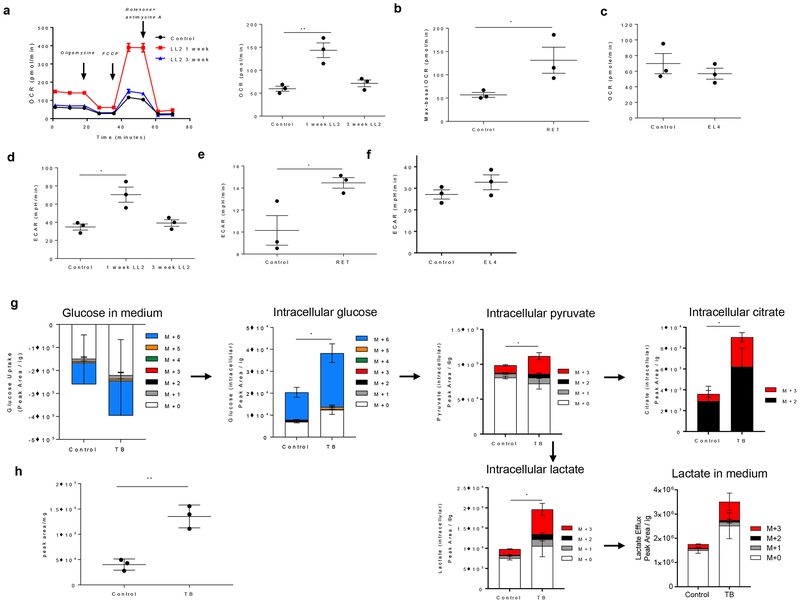
PM-LC have increased metabolic flux through oxidative phosphorylation and glycolysis and have more ATP than control neutrophils. **a, d**. Oxygen consumption rate (OCR) and extracellular acidification rate (ECAR) of neutrophils from control, one-week, and three-week LL2 TB mice. After 3 basal measurements, cells were treated with oligomycin, FCCP, and rotenone and antimycin. **a**. 18 measurements from 3 mice in each group were taken (left). Basal metabolic rates from 3 mice (right). **b**. Spare respiratory capacity (maximal rate post-FCCP treatment – basal rate) of PM-LC from 3 control and RET melanoma mice. **c**. Basal OCR of neutrophils from 3 control and 3 EL4 mice. **d**. ECAR of neutrophils from 3 control, 3 one week LL2 and 3 three week LL3 mice. **e,f**. Basal glycolytic rate of PM-LC from 3 control and 3 RET melanoma mice **(e)** and from 3 EL4 mice **(f)**. Individual results and mean and SD are shown. P values were calculated in two-sided Student’s t-test. * − p <0.05; **−p<0.01. **g**. Ex vivo tracing of ^13^C_6_-glucose metabolism in control neutrophils and PM-LC isolated from BM of one-week LL2 TB mice as determined by LC-MS. ^13^C labeling of pyruvate and lactate (M+3 for both) was used as a readout of glycolytic flux while labeling of citrate (M+2) was used as a readout of glucose flux into the TCA cycle. Changes in the isotopologue distribution (i.e. ^13^C labeling pattern) of the metabolites was analyzed using a grouped analysis, regular two-way ANOVA (Tukey correction) with multiple comparisons. * − p <0.05. Data are presented as mean peak areas and SD and are normalized to protein content. (n=3 individual mice per group). M+0, M+1, M+2, etc. indicate the number of ^13^C atoms in each metabolite (See Figure S7 for more details). Black arrows indicate the flow of carbon. **h**. LC-MS based measurement of intracellular ATP levels in control neutrophils and PM-LC. ATP levels were analyzed using an unpaired, two-tailed Student t-test. Data are presented as mean peak areas ± SD and are normalized to protein content. (n=3). P values were calculated in two-sided Student’s t-test. ** − p <0.01.

Next, we evaluated oxidative phosphorylation (OXPHOS) and glycolysis in BM neutrophils. We observed that BM PM-LCs from one-week LL2 mice had a markedly higher oxygen consumption rate (OCR) than control neutrophils, which is indicative of increased OXPHOS in these cells. In contrast, PMN-MDSCs from three-week LL2 mice had an OCR similar to control neutrophils (Fig. 6a). BM PM-LCs isolated from RET melanoma mice also demonstrated a significantly higher OCR compared to control neutrophils (Fig. 6b). In contrast, BM PMN-MDSCs from EL4 mice had OCR levels similar to that of control cells (Fig. 6c). PM-LCs from one-week LL2 mice also had a markedly higher extracellular acidification rate (ECAR) (Fig. 6d), which is indicative of increased glycolysis. BM PMN-MDSCs from three-week LL2 mice had ECAR similar to control cells. PM-LCs from RET melanoma mice also had a higher ECAR compared to control neutrophils (Fig. 6e). No differences were observed in BM PMN-MDSCs from EL4 mice (Fig. 6f). Thus, BM PM-LCs from the early stages of orthotopic lung cancer and from transgenic RET melanoma mice demonstrated increased OXPHOS and glycolysis compared to control neutrophils or PMN-MDSC.

We then performed metabolomics analysis of ^13^C_6_-glucose through glycolysis (Supplementary Fig. 5) in control neutrophils and PM-LCs from one-week tumor-bearing mice. BM PM-LC had higher uptake of glucose along with higher intracellular levels of pyruvate, citrate, and lactate compared to control neutrophils. They also secreted markedly more lactate (Fig. 6g). In addition, PM-LC had much higher rates of incorporation of the ^13^C atoms into pyruvate (M+3), lactate (M+3), and citrate (M+2) (Fig. 6g). The ^13^C-labeling patterns we observed in PM-LC strongly indicated that there was increased flux of glucose carbon through glycolysis and into the tricarboxylic acid (TCA) cycle in PM-LC. As a consequence of accelerated glycolysis and flux through the TCA cycle, PM-LC had significantly higher amounts of ATP (Fig. 6h). In contrast to PM-LC, metabolomics analysis of BM PMN-MDSCs from three-week s.c. LLC mice showed that PMN-MDSCs did not have higher ^13^C-labeling of pyruvate and lactate compared to neutrophils in control mice, nor were there any differences in the efflux of lactate into the media (Supplementary Fig. 6a). There were also no differences observed in the level of ATP in neutrophils and PMN-MDSCs (Supplementary Fig. 6b). These results were in agreement with the OCR and ECAR analyses where there were no differences in OXPHOS or glycolysis in PMN-MDSCs compared to control neutrophils. The increased metabolism of BM PM-LC was not associated with increased mitochondrial mass (Supplementary Fig. 4d).

Because neutrophils have few mitochondria and primarily use glycolysis to generate ATP^29^, we hypothesized that two major mechanisms could account for the increased glucose metabolic rate in PM-LCs: 1) an increase in the expression of glycolytic enzymes and 2) an increase in glucose uptake. PM-LC, but not PMN-MDSC, had increased expression of the Glut1 glucose transporter (Supplementary Fig. 6c,d), which may explain the increased glucose uptake by PM-LCs. We did not observe differences in the expression of major glycolytic enzymes between control neutrophils and PM-LC from RET melanoma mice (Supplementary Fig. 7a), which was consistent with the results obtained at RNA-Seq analysis. Thus, PM-LC had high glucose uptake, increased OXPHOS, increased TCA cycle flux, and increased glycolysis resulting in a substantially higher production of ATP compared to neutrophils from control mice, which may account for their increased migratory ability. This effect was absent in PMN-MDSCs.

### Increased spontaneous migration of PM-LC in cancer is regulated by ATP

**Figure 7.**
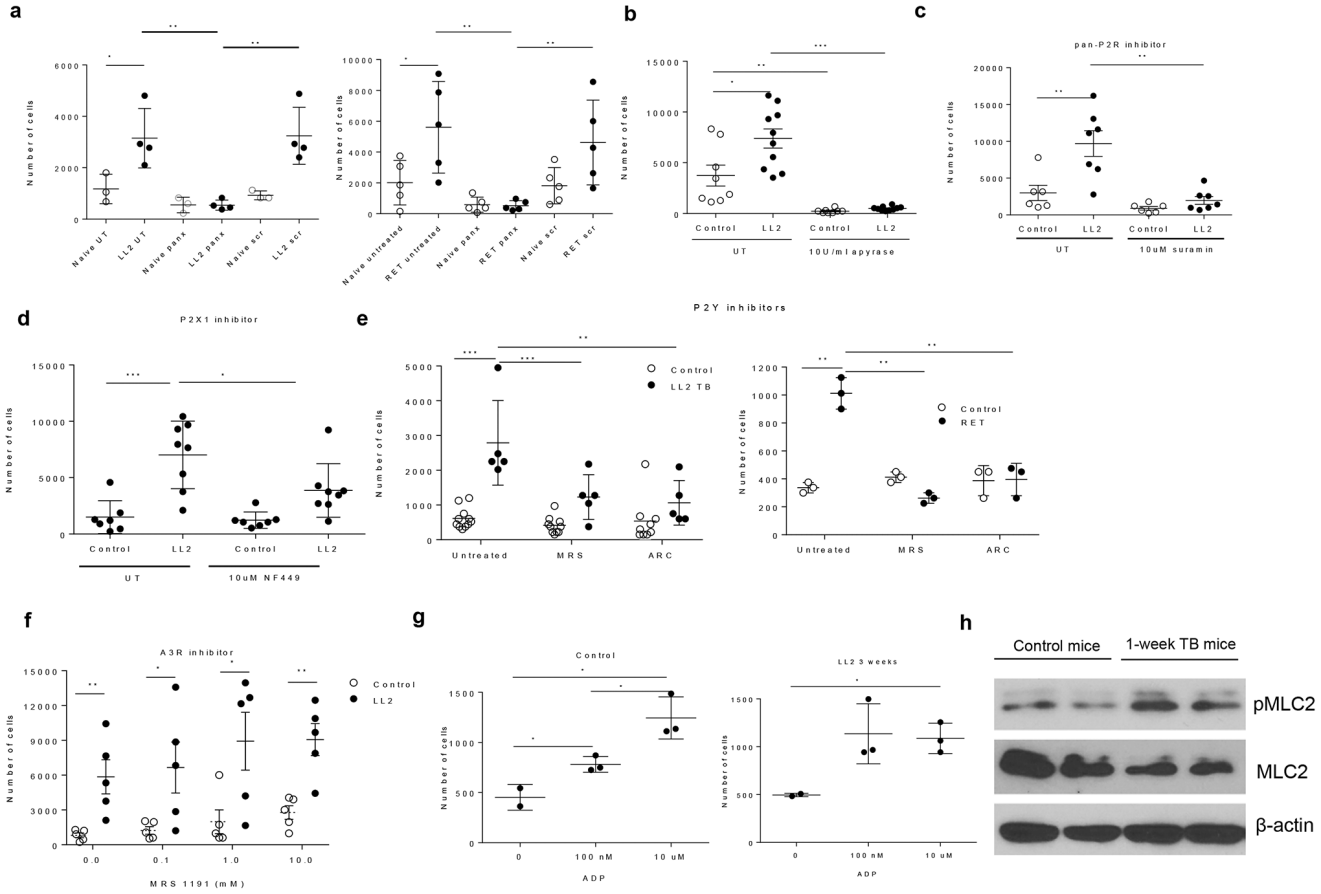
PM-LC spontaneous migration is dependent upon pannexin-1 hemichannels, extracellular ATP, and P2X and P2Y receptors. **a**. Transwell assays evaluating the ability of PM-LC from one-week LL2 mice (left) (n=3) and RET mice (right) (n=5) to spontaneously migrate upon pannexin-1 hemichannel inhibition (panx). **b**. Transwell assays evaluating the ability of neutrophils from control mice (n=8) and PM-LC from one-week LL2 mice (n=10) to spontaneously migrate upon apyrase treatment. (**c-f**) Transwell assays evaluating the ability of neutrophils from control mice (n=6) and PM-LC from one-week LL2 mice (n=7) to spontaneously migrate upon pan P2R inhibitor, P2XR, P2X1, P2Y, and A3R inhibition with suramin, NF449, MRS2179, AR-C118925XX, and MRS 1191, respectively. In addition, the ability of PM-LC from RET melanoma mice (n=3) to spontaneously migrate was also evaluated in the presence of P2Y inhibitors MRS2179 and AR-C118925XX. Individual values and mean and SD are shown. **g**. Migration of BM neutrophils from control mice (n=3) and PMN-MDSC from 3-week LL2 mice (n=3) in response to stimulation with different concentrations of ADP. Mean and SD are shown. P values are calculated in two-sided Student’s t-test. * − p <0.05; **−p<0.01; ***−p<0.001 **h**. pMLC2 in BM neutrophils isolated from naive mice and one-week LL2 TB mice. Experiments were performed three times with similar results.

ATP-mediated autocrine signaling can enhance gradient sensing and chemotaxis of neutrophils^30^. We hypothesized that BM PM-LC might use ATP-mediated autocrine signaling as a mechanism to support their enhanced spontaneous migration. ATP can be released from cells through mechanosensitive channels, such as pannexin-1 and connexin hemichannels^31, 32^. We examined the effect of disrupting ATP release from the cell on the ability of PM-LCs to spontaneously migrate. We inhibited pannexin-1 hemichannels using ^10^Panx (panx), a pannexin-1 mimetic inhibitory small peptide. This resulted in the abrogation of increased spontaneous migration exhibited by PM-LC from RET melanoma and one-week LL2 TB mice (Fig. 7a). The specificity of the effect was confirmed with scrambled ^10^Panx, which had no effect on the spontaneous migration of PM-LCs (Fig. 7a). To verify that spontaneous migration is truly dependent upon ATP, we treated cells with apyrase, an enzyme that catalyzes the hydrolysis of ATP. Upon apyrase treatment, the spontaneous migration of both control neutrophils and PM-LCs was completely abrogated (Fig. 7b).

There are several families of ectonucleotidases expressed on the cell surface of mammalian cells that can hydrolyze ATP to ADP, AMP, and adenosine, and each of these metabolites can signal through a distinct set of purinergic receptors^33^. Neutrophils have been reported to use the purinergic receptors P2X_1_, P2Y_1_, and P2Y_2_, which bind ATP, and A3, which binds adenosine, to amplify chemotactic signals^34, 35^. To determine if these receptors are involved in the spontaneous migration of PM-LCs, we treated cells with suramin, a pan-P2 receptor (pan-P2R) inhibitor and observed that spontaneous migration of PM-LCs was completely abrogated (Fig. 7c). To clarify the role of individual purinergic receptors, we treated cells with NF449, a P2X_1_-specific inhibitor. The ability of PM-LCs to spontaneously migrate was only partially (50%) inhibited (Fig. 7d). However, blocking the P2Y_1_ and P2Y_2_ receptors using the selective inhibitors MRS2179 and AR-C118925XX, respectively, completely abrogated the increased spontaneous migration (Fig. 7e). In contrast, blocking the A3 receptor with the MRS 1191 inhibitor did not affect the migration of PM-LC (Fig. 7f). These data indicated that increased production of ATP by PM-LC in TB mice was directly involved in the increased spontaneous migration of these cells in a paracrine fashion. If increased production of ATP and paracrine signaling was a major mechanism of increased spontaneous migration of PM-LC, then activating the purinergic receptors should increase the spontaneous migration of control neutrophils and PMN-MDSC. To test this hypothesis we treated these cells with ADP and observed significantly increased migration (Fig. 7g).

Next, we investigated the signaling pathways by which ATP could affect spontaneous neutrophil migration. Neutrophil migration requires polarization of the front and rear of the cell, processes that are coordinated by the Rho guanosine triphosphatases (GTPases) Rac, Cdc42 and RhoA^36^. Rac GTPase activation at the leading edge of the cell induces actin polymerization, and Cdc42 signaling can steer the direction of migration^37^. We evaluated actin polarization (F-actin) in neutrophils. Both, CXCL1 and fMLP stimulation resulted in substantial increase in F-actin. However, no differences between unstimulated and stimulated BM neutrophils from control and PM-LC from RET, KPC, or TRAMP TB mice were found (Supplementary Fig. 7b,c).

The rear of the cell generates the contractile force that is needed to overcome any cell adhesions and move the cell body forward. This process is regulated by activation of RHO-associated coiled-coil-containing protein kinase (ROCK), that phosphorylates Ser_19_ on the myosin light chain 2 (MLC2)^21^. We evaluated the target ROCK activity pMLC2 and found that PM-LC from one-week LL2 TB mice had substantial up-regulation of pMLC2 as compared to control neutrophils (Fig. 7h). Selective P2Y_2_ inhibitor AR-C118925XX abrogated up-regulation of pMLC2 in PM-LC (Supplementary Fig. 7e).

### Increased neutrophil migration is associated with increased tumor spread

**Figure 8.**
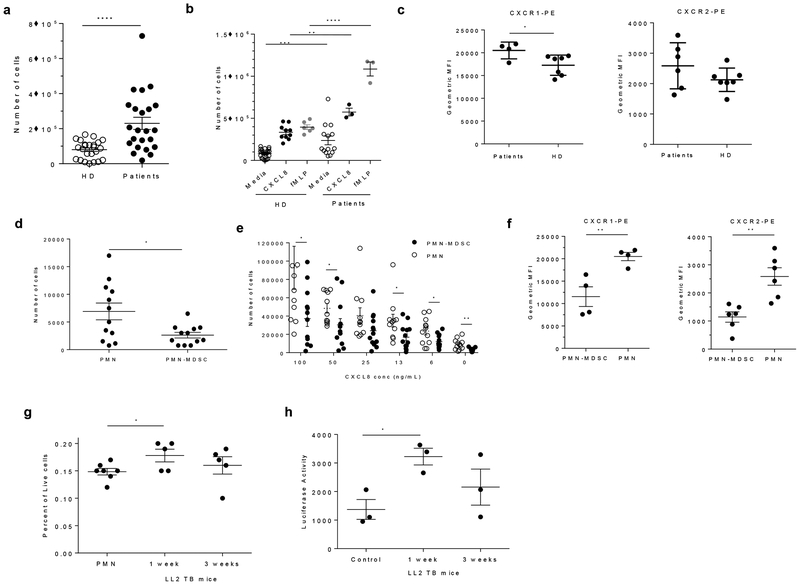
Neutrophils migration in cancer patients. Spontaneous (n=24 for both healthy donors and cancer patients groups) (**a**) or chemokine induced (for healthy donor n=24 for media, n=10 for CXCL8 stimulation, n=6 for fMLP stimulation; for cancer patients n= 14 for media, n=3 for CXCL8, and n=3 for fMLP); (**b)** migration of neutrophils from healthy individuals and cancer subjects. Individual results for each subject, mean and SD are shown. **c**. CXCR1 and CXCR2 expression in neutrophils from healthy individual (n=7) and cancer patients (n=4 for CXCR1 and n=6 for CXCR2) assessed by flow cytometry. Individual results for each subject, mean and SD are shown. **d, e**. Spontaneous (n=12) **(d)** and CXCL1 stimulated (n=11 for PMN and n=13 for PMN-MDSC) (**e**) migration of neutrophils and PMN-MDSC from the same cancer patients. **f**. Expression of chemokine receptors in human neutrophils. Indicated chemokine receptors were measured by flow cytometry on PMN-MDSC and neutrophils from cancer patients. N=4 for CXCR1 and n=6 for CXCR2. **g**. Percentage of CD45^−^CFSE^+^ LLC cells in lungs from naive mice 12 hours after intravenous injection. BM Ly6G^+^ neutrophils (PMN) from naïve (n=7), one week (n=5), or three-week (n=5) LL2 mice were injected 6 hours before tumor cells. **h**. IVIS-based analysis of ex-vivo luciferase activity in excised lungs from naive mice, which were injected i.v. with indicated BM Ly6G^+^ cells 5 hours before the i.v. injection of LL2 tumor cells. N=3. Detection was performed two weeks after the injection. In all panels mean and SD are shown. P values are calculated in two-sided Student’s t-test. * − p <0.05; **−p<0.01; ***−p<0.001; ****−p<0.0001.

Next, we evaluated the migratory behavior of neutrophils from cancer patients. Neutrophils were isolated from peripheral blood by negative selection and their response to CXCL8 (IL-8) and fMLP stimulation was assessed. Neutrophils from cancer patients demonstrated markedly higher spontaneous migration compared to neutrophils obtained from healthy donors (Fig. 8a). This was associated with more potent response to both CXCL8 and fMLP (Fig. 8b). Neutrophils from healthy donors and cancer patients had similar expression of the chemokine receptors CXCR1 and CXCR2 (Fig. 8c). We compared the motility of neutrophils and PMN-MDSC from the same patients. CD15^+^ PMN-MDSCs were isolated from the low density peripheral blood mononuclear cell (PBMC) using magnetic beads ^8^. CD15^+^ neutrophils were also isolated from the high-density fraction using magnetic beads^38^. Neutrophils exhibited substantially higher spontaneous CXCL8 stimulated migration than PMN-MDSCs from the same patient (Fig. 8d,e). PMN-MDSCs were found to have lower expression levels of CXCR1 and CXCR2 (Fig. 8f), which may account for their lower response to CXCL8. Despite the marked difference in migration, neutrophils and PMN-MDSC had similar level of actin polymerization (Supplementary Fig. 7d), a finding that is consistent with the results obtained in TB mice. These data suggest that neutrophils, but not PMN-MDSCs, in cancer patients have higher spontaneous and chemokine-induced migration than neutrophils from healthy donors.

We asked whether the increased spontaneous motility of PM-LC could facilitate tumor-cell seeding. To test this hypothesis, we performed two types of experiments where we assessed the role of neutrophils in promoting tumor-cell seeding of lungs after i.v. injection. In the first group of experiments, BM neutrophils from control mice, PM-LC from one-week LL2 mice and PMN-MDSC from three-week LL2 mice were injected i.v. into tumor-free recipients 5 hours before the injection of CFSE-labeled LL2 tumor cells. The presence of tumor cells in lungs was evaluated 12 hours later. Administration of PM-LC but not PMN-MDSC caused significantly higher seeding of tumor cells compared to administration of control neutrophils (Fig. 8g). In the second group of experiments, control neutrophils, PM-LCs, and PMN-MDSCs were injected i.v. 5 hours prior to administration of LL2-Luc tumor cells and tumor growth was assessed 14 days later by measuring luciferase luminescence. All mice developed tumors. Administration of PMN-MDSC slightly increased tumor burden. However, it was significantly larger after administration of PM-LC (Fig. 8h). Thus, PM-LC preferentially facilitate tumor-cell seeding, and it is possible that once PM-LC are in the tissue, these cells could facilitate tumor-cell metastasis (Supplementary Fig. 8).

## Discussion

Our study describes the association of the functional state of neutrophil activation in cancer with the ability of these cells to spontaneously migrate. It is likely that the population of neutrophils in tumor-bearing mice or cancer patients consists of classic neutrophils and a smaller population of pathologically activated immune-suppressive PMN-MDSCs. The abundance of PMN-MDSC depends on the stage and type of cancer^5^. PMN-MDSC usually accumulate in advanced stage cancer and are often barely detectable in the early stages. Although PMN-MDSC may be detected in BM, their suppressive activity in BM is much lower than that of cells in the spleen and tumor site^3^. Our data confirmed the presence of PMN-MDSC in the BM of mice with different s.c. (ectopic) tumors and in mice with advanced orthotopic lung cancer. However, immune-suppressive BM PMN-MDSC were not found in mice with GEM of cancer and in early stage orthotopic lung cancer. It is possible that the degree of the inflammation associated with the different tumor models plays a role. Ectopic tumors that require injection of a relatively large number of tumor cells s.c. are associated with local inflammation that may facilitate rapid development of PMN-MDSCs. The same phenomenon is associated with late stage orthotopic or spontaneous cancers where the mice have a large tumor burden. BM neutrophils from mice with early stage orthotopic lung cancer and GEM of cancer did not have immune-suppressive activity. However, BM neutrophils in these models had elevated expression of genes in the ER stress pathway, one of the characteristics of pathological activation of these cells in cancer ^39^. We found that these cells are markedly different from control BM neutrophils in their ability to spontaneously migrate. They also have increased expression of the genes associated with energy production, nucleic acid metabolism, increased OXPHOS and glycolytic rates, and considerably higher ATP compared to control neutrophils. Recently, to characterize pathologically activated myeloid cells that did not yet acquire potent suppressive activity, the term “MDSC-like” cells was introduced^8^. We used the term PMN-MDSC-like cells (PM-LC) to define the functional state of BM neutrophils from mice with early stage orthotopic lung cancer and GEM of cancer that did not have immune-suppressive activity.

Why do PM-LC have a potent ability to spontaneously migrate? In order to move, neutrophils must acquire spatial asymmetry. The crosstalk between the front and rear of the cell that maintain this polarization are poorly understood, but is believed to be maintained by coordinated Rho guanosine triphosphatase (GTPase) signaling between Rac, Cdc42 and RhoA^36^. In migrating neutrophils, leading edge protrusion (lamellipod) formation is caused by the polymerization of monomeric globular actin (G-actin) into filamentous actin (F-actin). The rear of the cell (uropod) generates the contractile force that is needed to overcome any cell adhesions and move the cell body forward. Activation of RhoA leads to the activation of RHO-associated coiled-coil-containing protein kinase (ROCK), that directly phosphorylates Ser19 on the regulatory light chain of myosin, MLC2^40^. We were focused on the two endpoints of the signaling cascades that result in neutrophil migration: F-actin polymerization and phosphorylation of MLC2. Although, as expected, F-actin was substantially increased in neutrophils in response to stimuli, no differences were observed between control neutrophils and PM-LC. In contrast, PM-LC had substantially higher level of pMLC2 implicating contractile force in the rear of cells as the driving force to support the increased spontaneous migration of PM-LC.

What drives increased migration of PM-LC? Our study demonstrated that BM PM-LC had increased ATP, whereas BM PMN-MDSCs had ATP levels similar to control neutrophils. This paralleled the changes in migratory capacity. The metabolism of MDSC in cancer is not well understood. In in vitro-generated MDSC, glycolysis increased concurrently with increased arginase I activity and activation of AMP-activated protein kinase, which can drive metabolism towards fatty acid oxidation (FAO)^41^. Tumor-infiltrating MDSC primarily used FAO and oxidative phosphorylation as their main metabolic pathway. In support of this finding, tumor-infiltrating MDSC increased fatty acid uptake and upregulated key FAO enzymes^42^. Our data demonstrate that increased metabolism and ATP in BM PM-LC precedes acquisition of the functional immunosuppressive characteristics of PMN-MDSC. Since BM PM-LC are the cells that preferentially migrate into tissues, our data are consistent with the observations on the metabolism of PMN-MDSC in tumors.

Recent studies have shown that neutrophils release ATP in response to chemokines and that autocrine signaling serves to amplify the chemotactic signal^30^. ATP is released through connexin and pannexin-1 hemichannels^31^ and bind to three major families of purinergic receptors: P1, P2X, and P2Y^43^. P2Y_2_ and A3 are important mediators of extracellular ATP, and with CD39, aid in the polarization of neutrophils by translocating to the leading edge of the cell^30,31^. P2X1 was implicated in RhoA activation and phosphorylation of MLC2 in human and murine neutrophils^44^. By blocking purinergic receptors and ATP hydrolysis in this study, we demonstrated a direct role for ATP to support increased spontaneous migration of PM-LC. At the same time, ADP, a ligand of purinergic receptors, substantially increased spontaneous migration of control neutrophils and PMN-MDSC, supporting the notion that ATP release may be sufficient to drive high PM-LC motility.

In line with our studies in mice, peripheral blood neutrophils from cancer patients exhibited dramatically higher spontaneous migration than neutrophils from healthy individuals. PMN-MDSC had substantially reduced migration, which was consistent with a previous report of a population of human PMN-MDSC with low migratory activity^23^. Bona-fide PMN-MDSCs are capable of migrating towards a chemokine gradient, such as one leading to the tumor site, but have very poor ability to migrate to uninvolved tissues. There is now ample evidence that neutrophils can acquire features of PMN-MDSC in tissues of tumor-bearing hosts^5^. PM-LC may represent the first step of pathologic activation of neutrophils in cancer. These cells possess a potent ability to migrate to distant sites in the absence of inflammation or tumor cells compared to control neutrophils or PMN-MDSC.

## Online methods

### Human subjects and samples.

Samples of peripheral blood were collected from patients at the Helen F. Graham Cancer Center. The study was approved by the Institutional Review Board (IRB) of the Christiana Care Health System at the Helen F. Graham Cancer Center and The Wistar Institute. All patients signed IRB approved consent forms. Samples were collected at Helen F. Graham Cancer Center from 18 patients with previously untreated stage II-IV non-small cell lung cancer (NSCLC) and 8 patients with stage III-IV head and neck cancer. This cohort includes 14 females and 12 males, aged 48–74 years.

### Mice models and mice treatments.

Female and male C57BL/6N CD45.1^+^ and female C57BL/6 CD45.2^+^ mice (aged 6–8 weeks) were purchased from Charles River Laboratories. Female OT-I TCR-transgenic mice (C57Bl/6-Tg(TCRaTCRb)1100mjb) (4–6 week old) and female Pmel TCR-transgenic mice (B6.Cg-Thy1^a^/Cy Tg(TcraTcrb)8Rest) (4–6 week old) were purchased from Jackson Laboratories. Female C57Bl/6 CD45.1^+^/2^+^ were generated by crossing a male CD45.1^+^ with a female CD45.2^+^. All the mice were housed in pathogen-free conditions and handled in accordance with the requirements of the guidelines for animal experiments. The research was approved by the Wistar Institutional Animal Care and Use Committee. For in vivo migration experiments, 1×10^6^ CD45.1^+^ Ly6G^+^ BM cells were mixed with 1×10^6^ CD45.2^+^ Ly6G^+^ BM cells in a total volume of 100 μL of PBS and injected intravenously in female C57Bl/6 CD45.1^+^/2^+^ mice.

### Cell lines.

EL4 lymphoma, LLC (Lewis Lung Carcinoma), CT26 colon carcinoma were purchased from ATCC. The LL2 (Lewis Lung Carcinoma) tumor cell line expressing luciferase was a gift from Rupal Ramakrishnan (H. Lee Moffitt Cancer Center). They were maintained in DMEM medium supplemented with 10% fetal bovine serum (FBS, Sigma-Aldrich) and 1% penicillin-streptomycin (ThermoFisher Scientific) at 37°C with 5% CO_2_. Tumors cells were injected intravenously at 5 × 10^4^ cells per mouse in a total volume of 100 μL of PBS.

## Antibodies and reagents are described in Supplemental Table 1.

### Mouse Neutrophil and PMN-MDSC Isolation.

Legs bones (tibias and femurs) cleaned of muscular tissues are cut at both ends. Bone marrow (BM) cells are then flushed out with a 25-gauge needle and a 5 mL syringe filled with a cold solution of PBS 1× (ThermoFisher Scientific), FBS 1%, EDTA 2 mM (ThermoFisher Scientific) (cell suspension buffer, CSB). Cell suspension is then filtered through a 70 μm strainer (Fischer Scientific) placed on a conical 50 mL Falcon tube. For spleens, the organ is put in a 70 μm strainer placed on a conical 50 mL Falcon tube and cut into small pieces. These pieces are then grinded against the cell strainer using the plunger of a 5 mL syringe and washed several times with cold CSB. Tubes are then centrifugated at 1500 rpm at 4°C, the supernatant is removed and red blood cells are lysed by resuspending the cell pellet in ammonium chloride lysis buffer for 5 minutes at room temperature. Cells are then washed with cold CSB, spin down and the pellet is resuspended in cold CSB and counted using Trypan blue (VWR). Single-cell suspensions from lungs were prepared using mouse lung dissociation kit (Miltenyi Biotec) according to the manufacturer’s recommendations with an additional red blood cell lysis step as described above. For BM Ly6G^+^ cells isolation, cells were labeled with biotinylated anti-Ly6G antibody (Miltenyi Biotec), incubated with streptavidin-coated microbeads (Miltenyi Biotec) and separated on MACS columns (Miltenyi Biotec).

### Transwell assays for migration and chemotaxis.

Unstimulated migration and chemotaxis was measured using a 3μm pore transwell system (Neuro Probe Inc. or Sigma-Aldrich). Advanced RPMI with CXCL1 (BioLegend) or fMLP (Sigma-Aldrich) was placed in the bottom of the transwell, as indicated. 0.1×10^6^ or 0.5×10^6^ cells were incubated with pannexin inhibitor (^10^Panx; Tocris), scrambled ^10^Panx (Tocris), pan-P2XR inhibitor suramin (R&D Systems), P2X1 inhibitor NF 449 (Tocris), A3R inhibitor MRS 1191 (Santa Cruz), or apyrase (New England Biolabs) in Advanced RPMI for 10 min prior to being plated on top of the Neuro Probe system or Sigma-Aldrich system filter, respectively, and placed at 37°C, 5% CO_2_ for 1 hour. 10μl media was taken from the bottom wells and counted by hemocytometer. The quantification of migrated neutrophils was done using the formula N = n × 10^4^ × 0.029 reflecting 29μl media in the bottom well.

### Seahorse assay.

Metabolic rates were determined using the Seahorse XF24 and XF96 Flux Analyzers (Seahorse Biosciences) following the manufacturer’s protocol. Briefly, the microplate was coated with 22.4 μg/ml Cell-Tak (Fisher) using 200mM sodium bicarbonate. 1.2×10^6^ or 0.4×10^6^ cells were seeded per well immediately after isolation in 50μl and 80μl of unbuffered RPMI (Sigma-Aldrich) for the XF24 and XF96 analyzers, respectively. The microplate was incubated for 30 min at 37°C to allow the cells to settle into a monolayer. Unbuffered RPMI was gently added to the wells without disturbing the monolayer to bring the assay volume to 675μl and 180μl for the XF24 and XF96 analyzer, respectively. The basal oxygen consumption rate (OCR) and extracellular acidification rate (ECAR) was measured, in addition to rate changes upon treatment with 5μM oligomycin (Sigma-Aldrich), 1μM FCCP (Sigma-Aldrich), and 0.75μM rotenone and 1μM antimycin A (Sigma-Aldrich).

### Metabolomics.

Neutrophils, PM-LC and PMN-MDSC cells were isolated from bone marrow and resuspended in our homemade culture medium which contains nutrients at physiological concentrations, and was previously described^45^. Cells were allowed to adjust to the new medium for 1 hr before 5.5mM of ^13^C_6_-glucose was spiked into the medium. Cells were incubated for a further 90 min with the stable isotope tracer and then harvested. For harvesting, cell pellets were washed twice in ice-cold PBS and extracted in a solution of LC-MS grade methanol, acetonitrile, and ultrapure water. Samples were centrifuged and the resulting cleared supernatant was transferred to a silanized MS vial and run by LC-MS. For analysis of nutrient uptake and efflux, the medium was removed and diluted 50-fold into extraction solution and vortexed on a thermomixer for 10 min at 4°C before being frozen at −80°C overnight. The extracted medium was thawed on ice the next day and following centrifugation, the cleared supernatants were transferred to silanized glass vials and either run immediately by LC-MS or stored at −80°C. Metabolite measurements were normalized based upon protein concentration determined from cell pellets. LC-MS metabolite flux analysis was performed on a Thermo Scientific Q Exactive Plus mass spectrometer equipped with a HESI II probe and coupled to a Shimadzu Nexera UHPLC system. Column and LC conditions are as described above. The mass spectrometer was operated in full-scan, polarity switching mode with the spray voltage set to 3.2 kV in positive ion mode and 2.5 kV in negative ion mode. The heated capillary was set at 275°C, the HESI probe at 350°C, and the S-lens RF level at 45. The gas settings for sheath, auxiliary and sweep were 40, 10 and 1 unit, respectively. The mass spectrometer was set to repetitively scan m/z from 70 to 1000, with the resolution set at 70,000, the AGC target at 1E6, and the maximum injection time at 80 ms. Metabolite identification and quantitation was performed with TraceFinder 3.1 software (Thermo Fisher Scientific).

### Real-time quantitative PCR.

Cells were lysed and RNA was isolated using the E.Z.N.A. total RNA purification kit (Omega Bio-Tek). Reverse transcription was performed using the High Capacity cDNA Reverse Transcription Kit (Applied Biosystems Inc.). Quantitative PCR was then performed using Sybr Green PCR Master Mix (Applied Biosystems Inc.) on an ABI 7500 Fast instrument. Primers are described in Supplemental Table 2.

### F-actin measurement.

Total bone marrow cells were stained with AQUA, CD11b-BV421, and Ly6G-APC. Cells were washed and stimulated with doses of CXCL1 and fMLP in Advanced RPMI for the time points indicated. Cells were immediately fixed and permeabilized using Cytofix/Cytoperm Solution (BD) and washed in Perm/Wash Buffer (BD) following the manufacturer’s protocol. Cells were stained with 1 unit of Phalloidin-AF488 (Thermo Fisher) for 20 min at 4°C, washed in Perm/Wash Buffer, run on a flow cytometer, and analyzed using FlowJo.

### Isolation of human neutrophils and PMN-MDSCs.

For isolation of total population of human neutrophils from healthy individuals and cancer subjects we used MACSxpress isolation kit (Miltenyi). For parallel isolation of PMN-MDSC and neutrophils, double density gradient of Histopaque-1077 and Histopaque-1119 (Sigma Aldrich) was used. PMN-MDSC were isolated from low density PBMC using CD15-beads (Miltenyi). Neutrophils were isolated from high density gradient also using CD15-beads. Neutrophil and PMN-MDSC purity was assessed by flow cytometry and was >95%.

### Flow cytometry.

All antibody incubations were performed for 15 minutes at 4°C in dark and centrifugations done at 1500 rpm at 4°C for 5 minutes, unless recommended otherwise by the manufacturer. Usually up to 1 × 10^6^ cells were incubated with Fc-block (BD Biosciences; clone 2.4G2; cat. no. 553142) in 50 μL of CBS, then washed in CBS and spin down before cell surface staining with additional antibodies. After the last incubation, cells were washed in CBS, spin down and resuspended in 400 μL of CBS before acquisition. Cells were run on LSRII flow cytometer (BD Biosciences) and data were analyzed by FlowJo (Tristar). For in vivo migration experiments analyses, the whole organ (spleen or lungs) was stained in the same way as described above except that up to 1 × 10^7^ cells were stained in 50 μL of CBS. Flow cytometry reporting summary can be found in Life Sciences Reporting Summary.

### RNA-seq.

Neutrophils were isolated using Ly6G beads with purity was >95%. RNA sequencing was performed using Illumina Hiseq 2500 platform (Illumina, San Diego, CA, USA). VAHTS Total RNA-Seq Library Preparation Kit was used for library preparation. Single-end read runs were used, with read lengths up to 50 bp in high output mode, 30M total read counts. Data was aligned using RSEM v1.2.12 software^46^ against mm10 genome and gene-level read counts and RPKM values on gene level were estimated for ensemble transcriptome. Samples with at least 80% aligned reads were analyzed. DESeq2^47^ was used to estimate significance between any two experimental groups. Overall changes were considered significant if passed FDR<5% thresholds with an additional threshold on fold change (fold>5) taken to generate the final gene set. Gene set enrichment analysis was done using QIAGEN’s Ingenuity® Pathway Analysis software (IPA®, QIAGEN Redwood City, www.qiagen.com/ingenuity) based on “Functions”, “Canonical Pathways”, “Upstream Regulators” and “Networks” options. RNA-seq data were deposited to GEO data repository, accession number GSE118366.

### Suppression assays.

After BM Ly6G+ cells isolation as described above, cells were plated in U-bottom 96-well plates in triplicates in complete RMPI without extra cytokines. They were co-cultured at different ratios with total splenocytes from Pmel or OT-1 transgenic mice in the presence of cognate peptides: OT-1, SIINFEKL; Pmel, EGSRNQDWL. Cells were incubated for 48 hours and then ^3^H thymidine (PerkinElmer) was added (1 μl/well) and incubated overnight. Samples were counted with a TopCount NXT instrument (PerkinElmer).

### In vivo tumor experiment.

In the short-term lung metastasis model, LLC were labelled with CFSE (BioLegend). 1×10^6^ labelled LLC cells were intravenously injected 5 hours after i.v injection of 1×10^6^ BM Ly6G^+^ cells isolated from naive mice, 1 week, or 3 weeks LL2 TB mice. At the time indicated, mice were sacrificed and lungs were collected after perfusion with PBS to eliminate circulating tumor cells. Lungs were cut into small pieces and digested using Lung Dissociation Kit according to the manufacture’s protocol (Miltenyi Biotec). Subsequently, the lungs were passed through a 70um cell strainer and subjected to red blood cell lysis using ammonium-chloride-potassium (ACK) buffer. Lung single cell suspensions were stained with CD45-APC-Cy7 (BD Biosciences).

For long-term lung metastasis model, 5×10^4^ LL2 cells were intravenously injected 5 hours after i.v injection of 2×10^6^ BM Ly6G^+^ cells isolated from naive mice, 1-week, or 3-weeks LL2 TB mice. Mice were sacrificed 14 days after injection and the tumor burden was analyzed. The luciferase signal was measured in excised lungs with an IVIS Spectrum imaging system (Caliper).

### Time-Lapse Migration Assay.

The wells of a 12-well plate were coated with 50 μg/ml fibronectin (Sigma-Aldrich, St. Louis, MO) and washed with PBS. 0.5 × 10^6^ cells were plated per well in Advanced RPMI (Thermo Fisher, Cat#: 12633–012) without FBS and placed in a 37°C, 5% CO_2_ incubator for 10–15 minutes to allow for cell attachment. The wells were gently washed with PBS 2× to remove non-adherent cells and Advanced RPMI was added to the wells. Cells were placed onto a motorized stage and observed using a Nikon Eclipse TE_300_ fluorescence microscope maintained in an environment of 37°C and 5% CO_2_. A 10× or 20× objective was used to capture images during the course of the time lapse. Images were captured every 30 seconds over the course of 15 minutes from at least four different fields of view.

### Measurement of cell trajectories and mean-squared displacements.

Cell movement was tracked using the ImageJ plugin Manual Tracking. ImageJ and the plugin are both freely available through the NIH website (http://rsbweb.nih.gov/ij/). The centroid of the cell was considered to represent the cell position. Time lapse microscopy was used, and images were taken every 1.5 minutes. The result was a series of (x,y) positions with time for each cell. The net displacement during the *i*th 1.5-minute increment, *D*_*i*_, was calculated by the difference of the position at the beginning and end of that time step. The mean-squared displacement, 〈*D*^2^(*t*)〉, over time was calculated using the method of non-overlapping intervals. Speed, S, can be considered as the total path length over time and persistence time, P, is the time a cell remains moving without changing direction. S and P were obtained by fitting these to the persistent random walk equation 〈*D*^2^(*t*)〉 = 2*S*^2^[*t* − *P*(1 − *e*^−*t*/*P*^)] where *t* is the time interval, using a non-linear least squares regression analysis ^48, 49^. The random motility coefficient (μ) was then calculated as $\mu =\frac{1}{2}S^{2}P$ ^50^

### Cytokine protein array.

Expression of cytokines in mouse sera was evaluated using mouse cytokine antibody array Panel A (ARY006, R&D Systems). Sera from one-week and three-week LL2 TB mice were mixed with a cocktail of biotinylated detection antibodies followed by streptavidin–labeled horseradish peroxidase and then visualized using chemiluminescence-based detection. Densities of the spots were quantified with Image J.

### Statistics.

Statistical analyses were done using two-tailed unpaired Student’s t tests. In mean-squared displacement two-way ANOVA with Bonferroni adjustment for multiple comparisons was used. Statistical tests were performed using GraphPad Prism version 7.0. P values of 0.05 were considered significant. RNAseq data were analyzed by RSEM v1.2.12 software, DESeq2, Ingenuity® Pathway Analysis software.

## Data availability

The data that support the findings of this study are available from the corresponding author upon request. Source data for each figure are provided in supplement. RNAseq data are deposited to GEO data repository, accession number GSE118366.

## Supplementary Material

1

2
