# Supplementary material for: Unique pattern of neutrophil migration and function during tumor progression
Source: Nat Immunol. Author manuscript; Available in PMC 2019 Apr 15. (PMC6195445; doi:10.1038/s41590-018-0229-5)
Supplement: 1 [file NIHMS1504334-supplement-1.pdf]

nature research

Corresponding author(s): Dmitry Gabrilovich

Reporting Summary

Nature Research wishes to improve the reproducibility of the work that we publish. This form provides structure for consistency and transparency in reporting. For further information on Nature Research policies, see Authors & Referees and the Editorial Policy Checklist.

Statistical parameters

When statistical analyses are reported, confirm that the following items are present in the relevant location (e.g. figure legend, table legend, main text, or Methods section).

n/a	Confirmed	
☐	☒	The exact sample size (*n*) for each experimental group/condition, given as a discrete number and unit of measurement
☐	☒	An indication of whether measurements were taken from distinct samples or whether the same sample was measured repeatedly
☐	☒	The statistical test(s) used AND whether they are one- or two-sided<br>*Only common tests should be described solely by name; describe more complex techniques in the Methods section.*
☒	☐	A description of all covariates tested
☐	☒	A description of any assumptions or corrections, such as tests of normality and adjustment for multiple comparisons
☐	☒	A full description of the statistics including central tendency (e.g. means) or other basic estimates (e.g. regression coefficient) AND variation (e.g. standard deviation) or associated estimates of uncertainty (e.g. confidence intervals)
☐	☒	For null hypothesis testing, the test statistic (e.g. *F*, *t*, *r*) with confidence intervals, effect sizes, degrees of freedom and *P* value noted<br>*Give P values as exact values whenever suitable.*
☒	☐	For Bayesian analysis, information on the choice of priors and Markov chain Monte Carlo settings
☒	☐	For hierarchical and complex designs, identification of the appropriate level for tests and full reporting of outcomes
☒	☐	Estimates of effect sizes (e.g. Cohen's *d*, Pearson's *r*), indicating how they were calculated
☐	☒	Clearly defined error bars<br>*State explicitly what error bars represent (e.g. SD, SE, CI)*

Our web collection on statistics for biologists may be useful.

Software and code

Policy information about availability of computer code

Data collection	Flow cytometry data was collected using BD FACSDiva software. Seahorse assay data was collected using Wave v2.4.
Data analysis	Metabolite identification and quantitation was performed with TraceFinder 3.1 (Thermo Fisher Scientific). Flow cytometry data was analyzed using FlowJo v9.6.2 (Tree Star, Inc.). Seahorse assay data was analyzed using Wave v2.4. Data analysis was also conducted using Prism v 7.0 (GraphPad Software).

For manuscripts utilizing custom algorithms or software that are central to the research but not yet described in published literature, software must be made available to editors/reviewers upon request. We strongly encourage code deposition in a community repository (e.g. GitHub). See the Nature Research guidelines for submitting code & software for further information.

Data

Policy information about availability of data

All manuscripts must include a data availability statement. This statement should provide the following information, where applicable:

- Accession codes, unique identifiers, or web links for publicly available datasets
- A list of figures that have associated raw data
- A description of any restrictions on data availability

The data that support the findings of this study are available from the corresponding author upon reasonable request.

Field-specific reporting

Please select the best fit for your research. If you are not sure, read the appropriate sections before making your selection.

☒ Life sciences ☐ Behavioural & social sciences ☐ Ecological, evolutionary & environmental sciences

For a reference copy of the document with all sections, see nature.com/authors/policies/ReportingSummary-flat.pdf

Life sciences study design

All studies must disclose on these points even when the disclosure is negative.

Sample size	Samples sizes were determined based off of published studies that utilize murine and human samples.
Data exclusions	*Describe any data exclusions. If no data were excluded from the analyses, state so OR if data were excluded, describe the exclusions and the rationale behind them, indicating whether exclusion criteria were pre-established.*
Replication	*Describe the measures taken to verify the reproducibility of the experimental findings. If all attempts at replication were successful, confirm this OR if there are any findings that were not replicated or cannot be reproduced, note this and describe why.*
Randomization	Not relevant to the study
Blinding	Not relevant to the study

Reporting for specific materials, systems and methods

Materials & experimental systems

n/a	Involved in the study	
☒	☐	Unique biological materials
☐	☒	Antibodies
☐	☒	Eukaryotic cell lines
☒	☐	Palaeontology
☐	☒	Animals and other organisms
☒	☐	Human research participants

Methods

n/a	Involved in the study	
☒	☐	ChIP-seq
☐	☒	Flow cytometry
☒	☐	MRI-based neuroimaging

Antibodies

Antibodies used	BD Biosciences: CD45.1-FITC (clone A20; catalog #: 553775), CD45.2-APC-Cy7 (clone: 104; catalog #: 560694), Ly6G-PE (clone: 1A8; catalog #: 551461), CD14-APC-Cy7 (clone: MϕP-9, catalog #: 557831); Ly6G-APC (clone: 1A8; catalog #: 560599); Mouse IgG1, κ isotype control-FITC (clone: MOPC-21, catalog #: 555909); CXCR2-FITC (clone: 6C6; catalog #: 551126);<br>BiolLegend: CD15-PerCP-Cy5.5 (clone: SSEA-1; catalog #: 323020); CXCR1-PE (clone: None; catalog #: 320608); Mouse IgG2b, κ isotype control-PE (clone: MPC-11; catalog #: 400314); CXCR4-PE (clone: 306505; catalog #: None); Mouse IgG2a, κ isotype control-PE (clone: MOPC-173; catalog #: 400214); CD11b-BV421 (clone: M1/70; catalog #: 101236)<br>R&D Systems: CXCR2-PE (clone: 242216; catalog #: FAB2164P); CXCR1-PE (clone: 1122A; catalog #: FAB8628P)<br>Novus Biologicals: Glut1 (clone: None; catalog #: NB110-39113); Glut4 (clone: None; catalog #: NBP1-49533)
Validation	Validation for primary antibodies can be found on the respective datasheets for each antibody.

Eukaryotic cell lines

Policy information about cell lines

Cell line source(s)	EL4, LLC, and CT26 cell lines were obtained from ATCC. The LL2 cell line expressing luciferase was a gift from Rupal Ramakrishnan (H. Lee Moffitt Cancer Center). The 4662 line
Authentication	None of the cell lines were authenticated.
Mycoplasma contamination	The cell lines tested negative for mycoplasma.

Commonly misidentified lines
(See ICLAC register)

None

Animals and other organisms

Policy information about studies involving animals; ARRIVE guidelines recommended for reporting animal research

Laboratory animals	Female and male C57BL/6 CD45.1+ and female C57BL/6 CD45.2+ mice (aged 6–8 weeks) were purchased from Charles River Laboratories. Female C57Bl/6 CD45.1+/2+ were generated by crossing a male CD45.1+ with a female CD45.2+. Female OT-I TCR-transgenic mice (C57Bl/6-Tg(TCRaTCRb)1100mjb) (4–6 week old) and female Pmel TCR-transgenic mice (B6.Cg-Thy1a/Cy Tg(TcraTcrb)8Rest) (4–6 week old) were purchased from Jackson Laboratories.
Wild animals	The study did not involve wild animals.
Field-collected samples	The study did not involve samples collected from the field.

Flow Cytometry

Plots

Confirm that:

☒ The axis labels state the marker and fluorochrome used (e.g. CD4-FITC).

☒ The axis scales are clearly visible. Include numbers along axes only for bottom left plot of group (a 'group' is an analysis of identical markers).

☒ All plots are contour plots with outliers or pseudocolor plots.

☒ A numerical value for number of cells or percentage (with statistics) is provided.

Methodology

Sample preparation	Murine cells from the bone marrow were obtained by removing the muscle and connective tissue from the femurs and tibia of euthanized mice. The tibia and femur were cut at both ends and the bone marrow was flushed from the bones with a cold solution of PBS 1x (ThermoFisher Scientific), FBS 1%, EDTA 2 mM (ThermoFisher Scientific) (cell suspension buffer, CSB) using a 25-gauge needle (Fisher Scientific, Cat#: 14-826AA), and red blood cells were lysed using Ammonium-Chloride-Potassium (ACK) lysing buffer. Cells were filtered through 70µm cell strainer (Fisher Scientific, Cat#: 087712) and resuspended in MACS buffer (0.5% heat-inactivated FBS and 2mM EDTA in PBS, filtered) for further use.<br>Murine cells from the blood were obtained by<br>Murine spleens were put in a 70 µm strainer placed on a conical 50 mL Falcon tube and cut into small pieces. These pieces were then grinded against the cell strainer using the plunger of a 5 mL syringe and washed several times with cold CSB. Tubes were centrifugated at 1500 rpm at 4°C, the supernatant was removed and red blood cells were lysed by resuspending the cell pellet in ACK lysis buffer for 5 minutes at room temperature. Cells are then washed with cold CSB, spin down and the pellet is resuspended in cold CSB and counted using Trypan blue (VWR).<br>Murine single-cell suspensions from lungs were prepared using mouse lung dissociation kit (Miltenyi Biotec) according to the manufacturer's recommendations with an additional red blood cell lysis step as described above.<br>Human neutrophils from healthy individuals and cancer subjects were isolated using the MACSxpress isolation kit (Miltenyi). For parallel isolation of PMN-MDSC and neutrophils, double density gradient of Histopaque-1077 and Histopaque-1119 (Sigma Aldrich) was used. PMN-MDSC and neutrophils were isolated from the low density PBMCs, and high-density gradient, respectively, using CD15-beads (Miltenyi), per manufacturer's protocol.
Instrument	Data were collected on either BD Biosciences LSRII 14- (Model #:) or 18-color (Model #:) instruments.
Software	BD FACSDiva software was used to collect the data. FlowJo software (Tree Star, Inc.) was used to analyze the data.
Cell population abundance	Not applicable
Gating strategy	For the CXCR1/CXCR2, mitochondrial mass, Glut1/3/4, ROS, and F-actin experiments on murine samples, cells gated from the SSC-A/FSC-A plot were then plotted against the cell viability marker (AQUA live-dead) and negative cells were selected. These cells were plotted on FSC-A/CD11b and CD11b-positive cells were selected. These cells were plotted on Ly-6G/Ly-6C and the Ly6G-positive cells were selected. A histogram of the marker in question yieed the MFI for that marker in this population. For the in vivo migration experiments, splenic or lung cells gated from the SSC-A/FSC-A plot were then plotted against Ly6G, and Ly6G-positive cells were selected. Cells were then plotted on CD45.1/CD45.2 to identify the population of CD45.1- or CD45.2-positive cells distinct from the double-positive CD45.1/CD45.2 recipient Ly6G-positive cells. For human samples, cells from the SSC-A/FSC-A plot were plotted against the cell viability marker (AQUA live-dead) and negative cells were selected. These cells were plotted on FSC-A/CD11b and CD11b-positive cells were selected. These cells were plotted on SSC-A/CD14, and CD14-negative cells were selected. These cells were plotted on SSC-A/CD15 and CD15-positive cells were selected. A histogram of the marker in question yielded the MFI for that marker in this population.

☐ Tick this box to confirm that a figure exemplifying the gating strategy is provided in the Supplementary Information.
