# Supplementary material for: Unique pattern of neutrophil migration and function during tumor progression
Source: Nat Immunol. Author manuscript; Available in PMC 2019 Apr 15. (PMC6195445; doi:10.1038/s41590-018-0229-5)
Supplement: 2 [file NIHMS1504334-supplement-2.pdf]

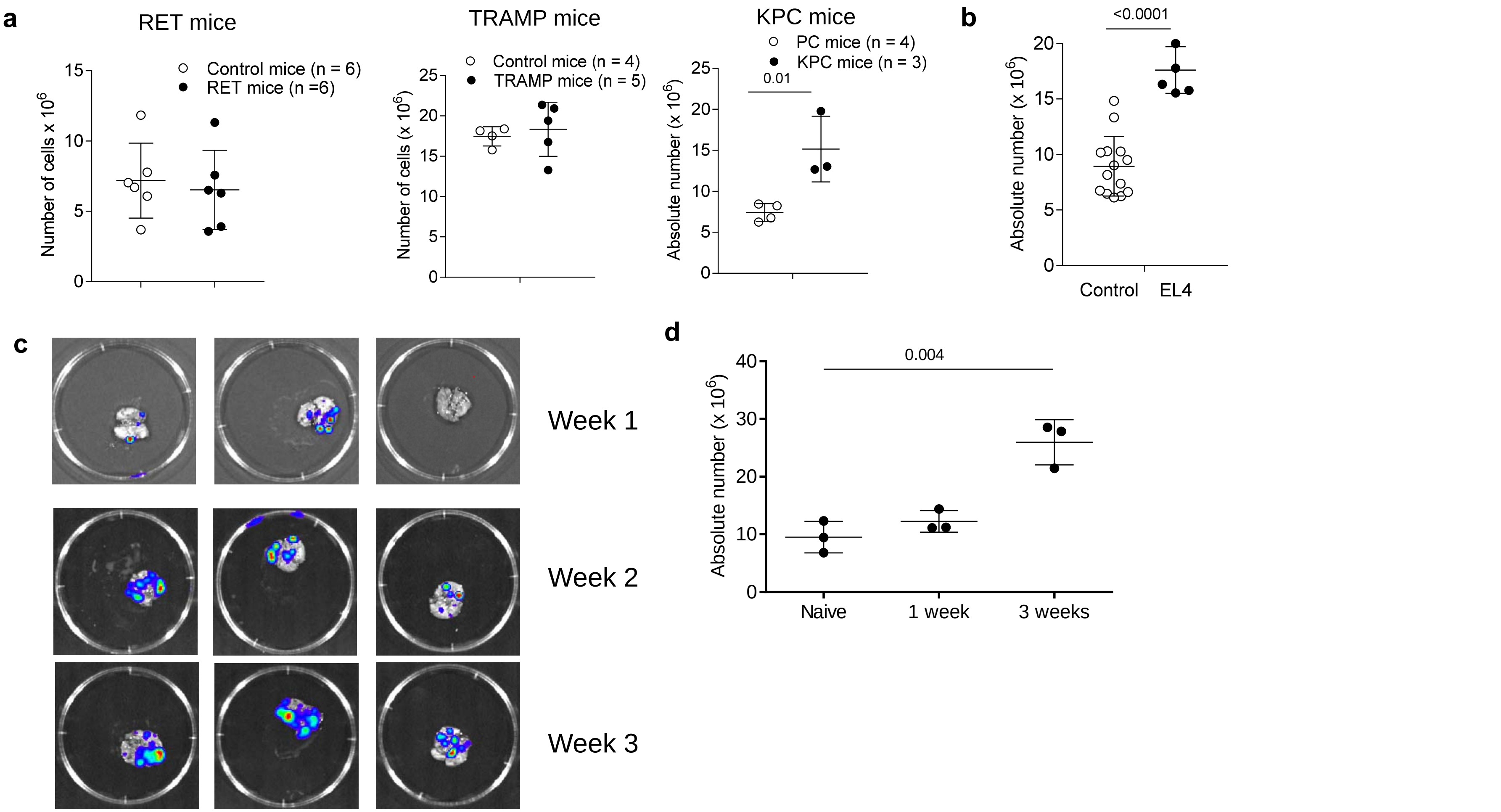

Supplemental Figure 1. The total number of CD11b^{+}Ly6C^{low}Ly6G^{+} neutrophils in BM of tumor-bearing mice. a. GEM models (n=6 for RET mice, n= 5 for TRAMP mice, n=3 for KPC mice), **b.** ectotopic s.c. models (n=5 for EL4 mice), **c.** Example of luciferase activity in lungs extracted from mice injected i.v. with 5x10^{4} LL2 tumor cells. **d.** orthotopic LL2 model (n=3). Individual results, mean and SD are shown. P values were calculated in two-sided Student's t-test.

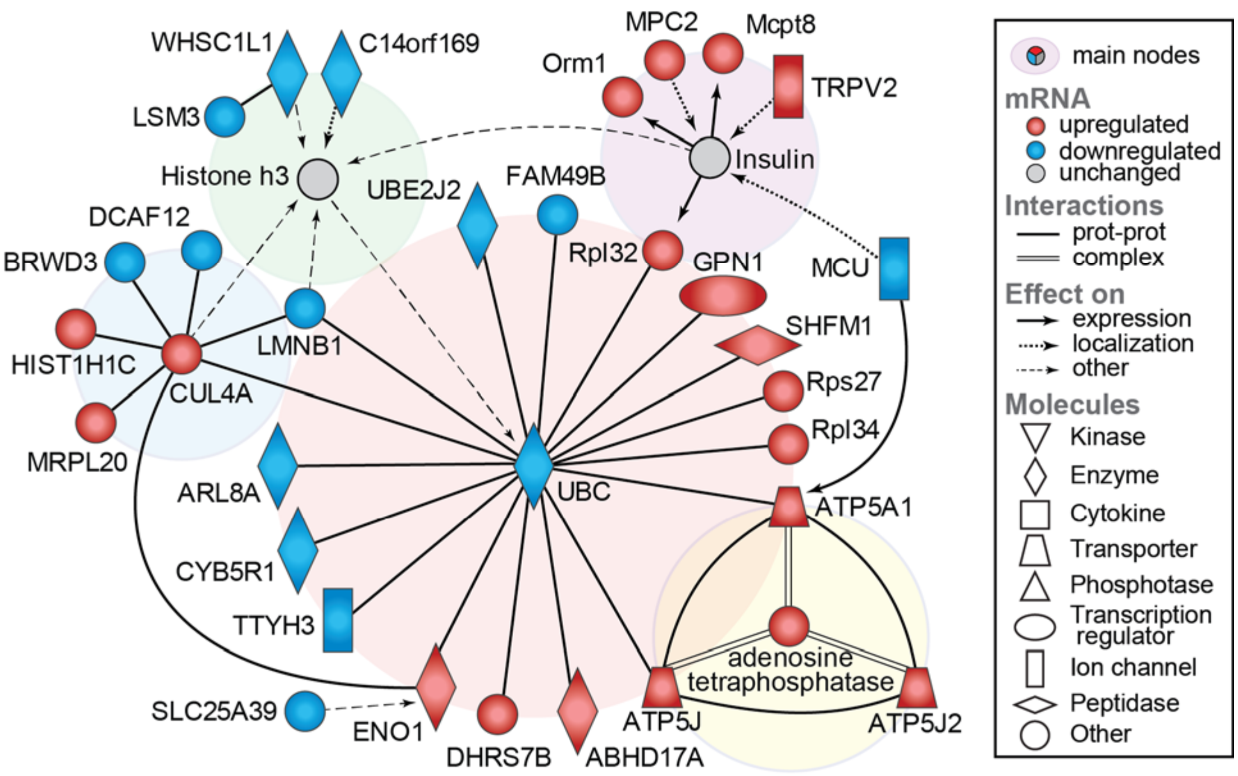

Supplemental Figure 2. Network Ingenuity Pathway Analysis of genes changed at week 1 compared to naïve. Top network (score=53) annotated with Top Diseases and Functions as "Energy Production, Nucleic Acid Metabolism, Small Molecule Biochemistry" is shown.

a

Week 1 vs. Control

w1/N	w3/N	w3/1	w1/N	w3/N	w3/1	Naïve		w1		w3			
fold	fold	fold	fdr	fdr	fdr	1	2	1	2	1	2	Symbol	Description
2.87	1.97	-1.46	0%	0%	0%							Pglyrp1	peptidoglycan recognition protein 1
2.39	1.43	-1.68	0%	0%	0%							Arhgap31	Rho GTPase activating protein 31
2.22	1.29	-1.71	0%	28%	0%							1700008O03Rik	RIKEN cDNA 1700008O03 gene
2.04	1.22	-1.67	0%	40%	0%							Kcnmb4	potassium large conductance calcium-activated channel
1.99	1.10	-1.82	0%	92%	0%							Il11	interleukin 11
1.98	1.26	-1.58	0%	20%	0%							Mbtps2	membrane-bound transcription factor peptidase, site 2
1.89	1.90	1.01	0%	0%	100%							Rpl6	ribosomal protein L6
1.73	1.76	1.02	0%	0%	99%							Rps29	ribosomal protein S29
-1.60	-1.27	1.26	0%	1%	2%							Ubc	ubiquitin C
-1.60	-1.11	1.45	0%	66%	0%							Lrrc17	leucine rich repeat containing 17
-1.63	1.30	2.12	0%	1%	0%							Ddx6	DEAD (Asp-Glu-Ala-Asp) box polypeptide 6
-1.71	-1.46	1.17	0%	0%	27%							Usf2	upstream transcription factor 2
-1.71	-1.02	1.68	0%	100%	0%							Slc16a3	solute carrier family 16 (monocarboxylic acid transporters)
-1.77	1.13	2.00	0%	52%	0%							Gnb2	guanine nucleotide binding protein (G protein), beta 2
-1.80	-1.27	1.42	0%	5%	0%							Lypla2	lysophospholipase 2
-1.82	-1.27	1.43	0%	7%	0%							Rrbp1	ribosome binding protein 1
-1.85	-1.35	1.37	0%	2%	1%							Gsk3b	glycogen synthase kinase 3 beta
-1.89	1.21	2.29	0%	15%	0%							Rassf5	Ras association (RalGDS/AF-6) domain family member 5
-1.91	-1.37	1.40	0%	0%	0%							Lmnb1	lamin B1
-1.97	-2.92	-1.49	0%	0%	0%							St3gal5	ST3 beta-galactoside alpha-2,3-sialyltransferase 5
-2.05	-1.82	1.13	0%	0%	79%							Ets2	E26 avian leukemia oncogene 2, 3' domain
-2.24	-1.50	1.49	0%	0%	0%							Rraga	Ras-related GTP binding A
-2.34	-1.77	1.33	0%	0%	2%							Fus	fused in sarcoma
-2.38	-1.05	2.25	0%	100%	0%							Tsc22d3	TSC22 domain family, member 3
-2.76	-1.58	1.74	0%	1%	0%							Dbt	dihydrolipoamide branched chain transacylase E2
-2.78	-1.35	2.07	0%	12%	0%							Akna	AT-hook transcription factor
-2.99	-2.04	1.46	0%	0%	17%							Fhod1	formin homology 2 domain containing 1
-3.10	-2.24	1.39	0%	0%	19%							Spag7	sperm associated antigen 7
-4.41	-2.77	1.59	0%	0%	10%							Grm1	glutamate receptor, metabotropic 1
-4.61	-2.21	2.09	0%	0%	0%							Epha5	Eph receptor A5

b

Week 3 vs. Control

w1/N	w3/N	w3/1	w1/N	w3/N	w3/1	Naïve		w1		w3			
fold	fold	fold	fdr	fdr	fdr	1	2	1	2	1	2	Symbol	Description
1.20	21.24	17.68	88%	0%	0%							BC100530	cDNA sequence BC100530
-1.03	20.71	21.27	100%	0%	0%							Stfa2	stefin A2
-1.45	15.39	22.37	10%	0%	0%							Prok2	prokineticin 2
1.67	14.24	8.51	17%	0%	0%							Stfa3	stefin A3
-1.13	13.24	15.01	100%	0%	0%							Fignl2	fidgetin-like 2
-1.21	9.82	11.89	100%	0%	0%							0610040J01Rik	RIKEN cDNA 0610040J01 gene
1.10	7.76	7.06	100%	0%	0%							Cd38	CD38 antigen
-1.63	7.48	12.23	15%	0%	0%							Cd244	CD244 natural killer cell receptor 2B4
-1.00	5.81	5.82	100%	0%	0%							Asprv1	aspartic peptidase, retroviral-like 1
1.06	5.75	5.43	100%	0%	0%							Stfa1	stefin A1
1.54	5.53	3.59	0%	0%	0%							Stfa2l1	stefin A2 like 1
-1.30	4.97	6.47	34%	0%	0%							Socs3	suppressor of cytokine signaling 3
-1.27	4.57	5.80	66%	0%	0%							6330416G13Rik	RIKEN cDNA 6330416G13 gene
-1.16	4.53	5.24	91%	0%	0%							Il4ra	interleukin 4 receptor, alpha
1.11	4.24	3.80	100%	0%	0%							Atp13a2	ATPase type 13A2
-1.09	4.05	4.44	100%	0%	0%							Cd14	CD14 antigen
1.46	3.65	2.49	0%	0%	0%							Ifitm6	interferon induced transmembrane protein 6
-1.18	3.42	4.05	54%	0%	0%							Ctnnbip1	catenin beta interacting protein 1
-1.37	3.39	4.64	8%	0%	0%							Zfp36l1	zinc finger protein 36, C3H type-like 1
-1.18	3.26	3.86	54%	0%	0%							Spata13	spermatogenesis associated 13
-1.42	3.24	4.59	4%	0%	0%							Ddi2	DNA-damage inducible protein 2
1.09	2.79	2.57	100%	0%	0%							Tgfbi	transforming growth factor, beta induced
-1.31	2.77	3.62	4%	0%	0%							Snx1	sorting nexin 1
1.10	2.76	2.52	100%	0%	0%							Emilin2	elastin microfibril interfacer 2
-1.13	2.67	3.03	85%	0%	0%							Ube2h	ubiquitin-conjugating enzyme E2H
1.12	2.66	2.38	95%	0%	0%							S100a6	S100 calcium binding protein A6 (calcyclin)
-1.24	-3.40	-2.75	39%	0%	0%							Atg16l2	autophagy related 16-like 2 (S. cerevisiae)
-1.71	-4.13	-2.41	0%	0%	0%							Il1rn	interleukin 1 receptor antagonist
-1.10	-4.71	-4.28	100%	0%	0%							Acta2	actin, alpha 2, smooth muscle, aorta
-1.39	-6.12	-4.41	24%	0%	0%							Rasl11b	RAS-like, family 11, member B

Expression fold vs mean

≤ -2	-1.5	-1.2	-1.1	±1	1.1	1.2	1.5	≥ 2

Supplemental Figure 3. Top 30 differentially expressed known genes. a. At week 1 vs Control. **b**. At week 3 vs Control. Insignificant fold changes with nominal p>0.05 are shown in white.

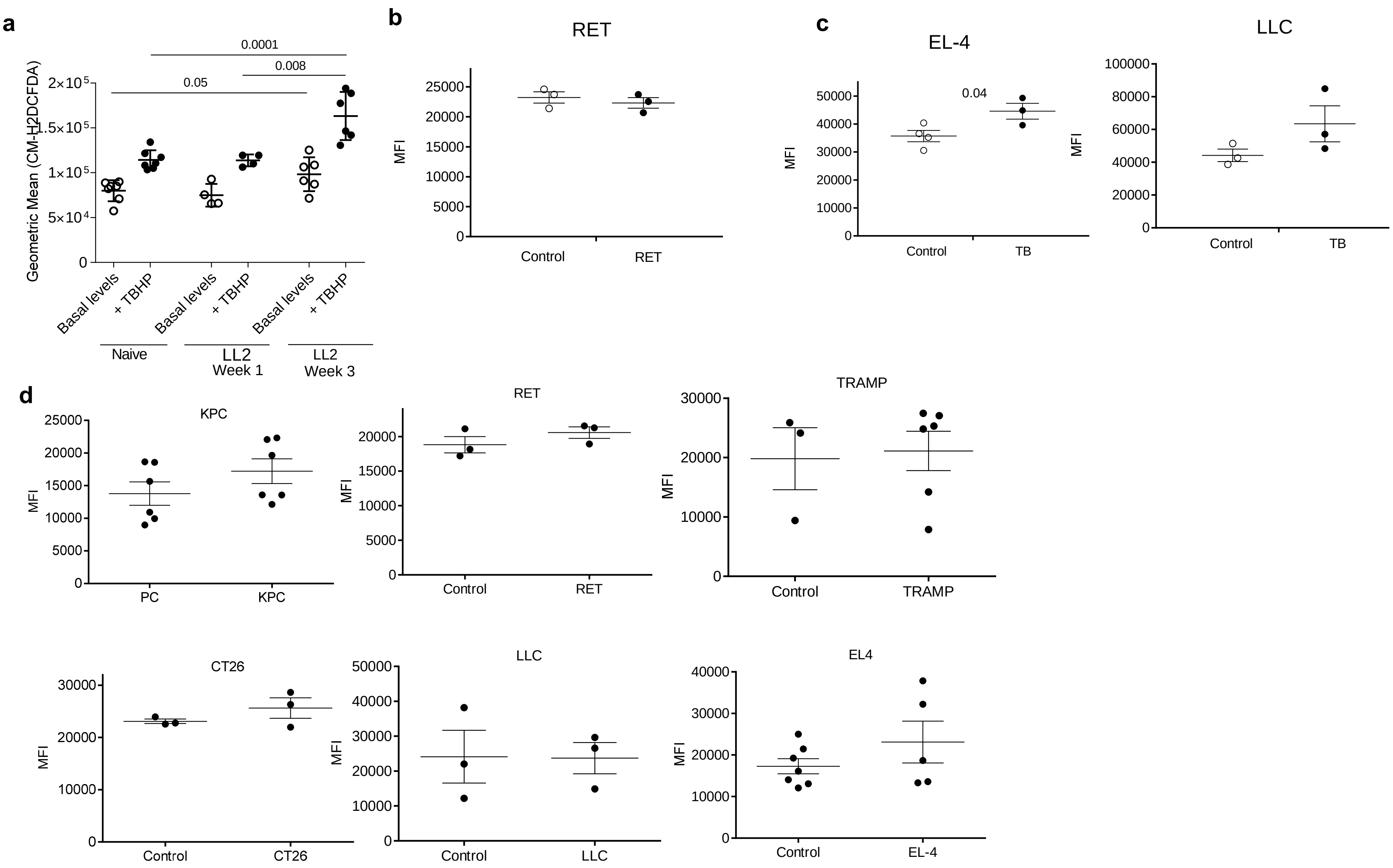

Supplemental Figure 4. Cytoplasmic ROS level in neutrophils. Spontaneous and stimulated (TBHP) ROS level in BM neutrophils from naïve (n=7), one- (n=4) and three-week (n=6) LL2 mice (**a**), RET melanoma (n=3) **(b)**, EL4 (n=3) and LLC (n=3) mice **(c)**. Individual results for each mouse, mean and SD are shown. P values in two-sided Student's t-test are shown. **d.** Mitochondrial mass of neutrophils and PMN-MDSC. Mitochondrial mass was measured using MitoTracker^{TM} Green-FM and flow cytometry in BM neutrophils from indicated tumor models. KPC (n=6), RET (n=3), TRAMP (n=6), CT26 (n=3), LLC (n=3), EL4 (n=5). Individual results for each mouse, mean and SD are shown.

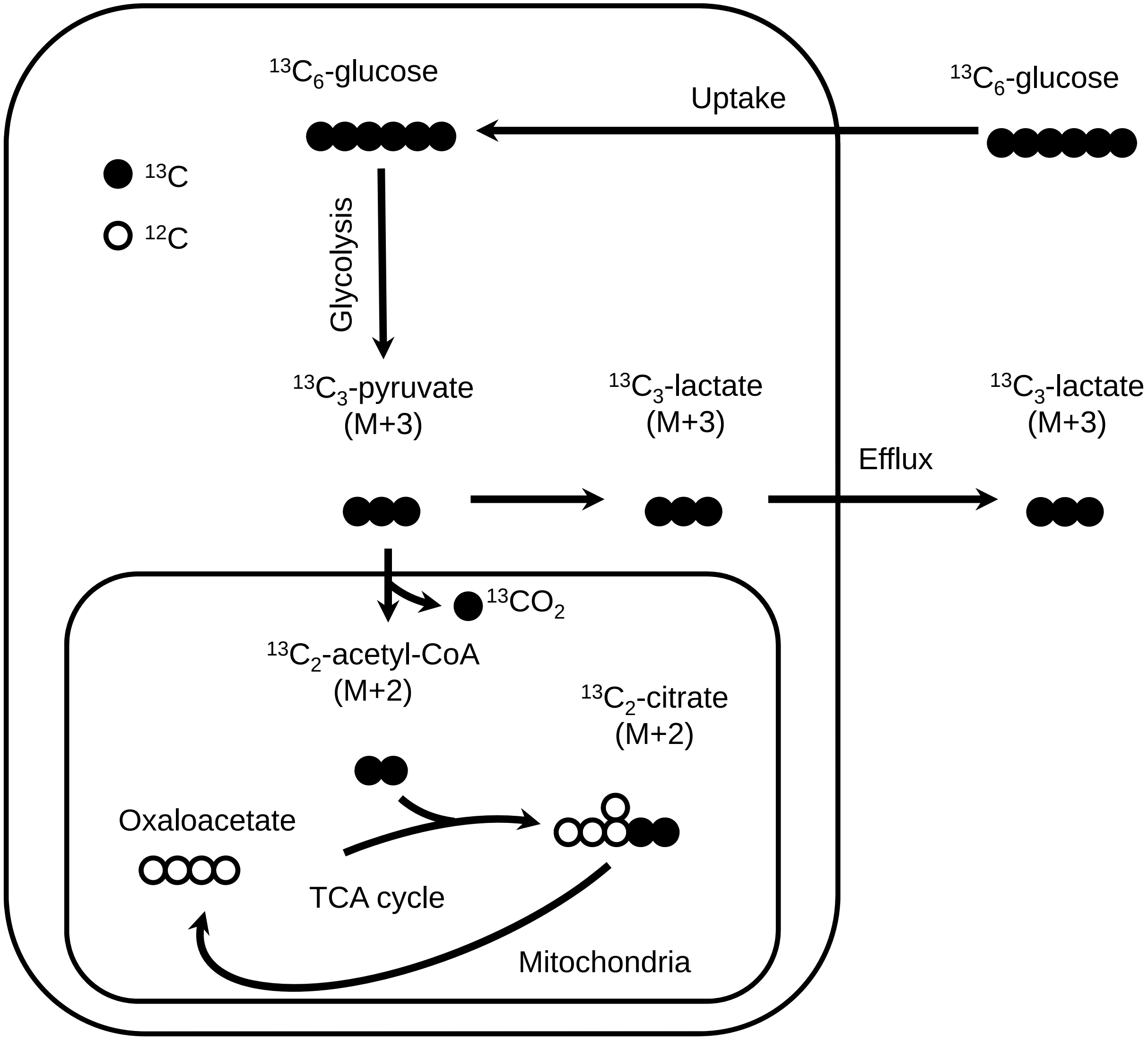

Supplemental Figure 5. Illustration of the flow of $^{13}C_6$-glucose through glycolysis and into the TCA cycle. The black circles represent carbon-13, the clear circle represent carbon-12. The six carbons of glucose are broken into two molecules of three carbons each half through glycolysis. Hence, the appearance of pyruvate containing three carbon-13 atoms (i.e. M+3) is a readout of glycolysis. Subsequently, if pyruvate is converted to lactate, the lactate will also have all three carbons labeled (M+3). However, if pyruvate enters the mitochondria, pyruvate dehydrogenase catalyzes its decarboxylation (loss of CO_2) thereby creating a two carbon-13 labeled acetyl-CoA molecule. The two carbons of acetyl-CoA are then used in the synthesis of citrate (M+2) and can be used as a readout of glucose flux into the TCA cycle.

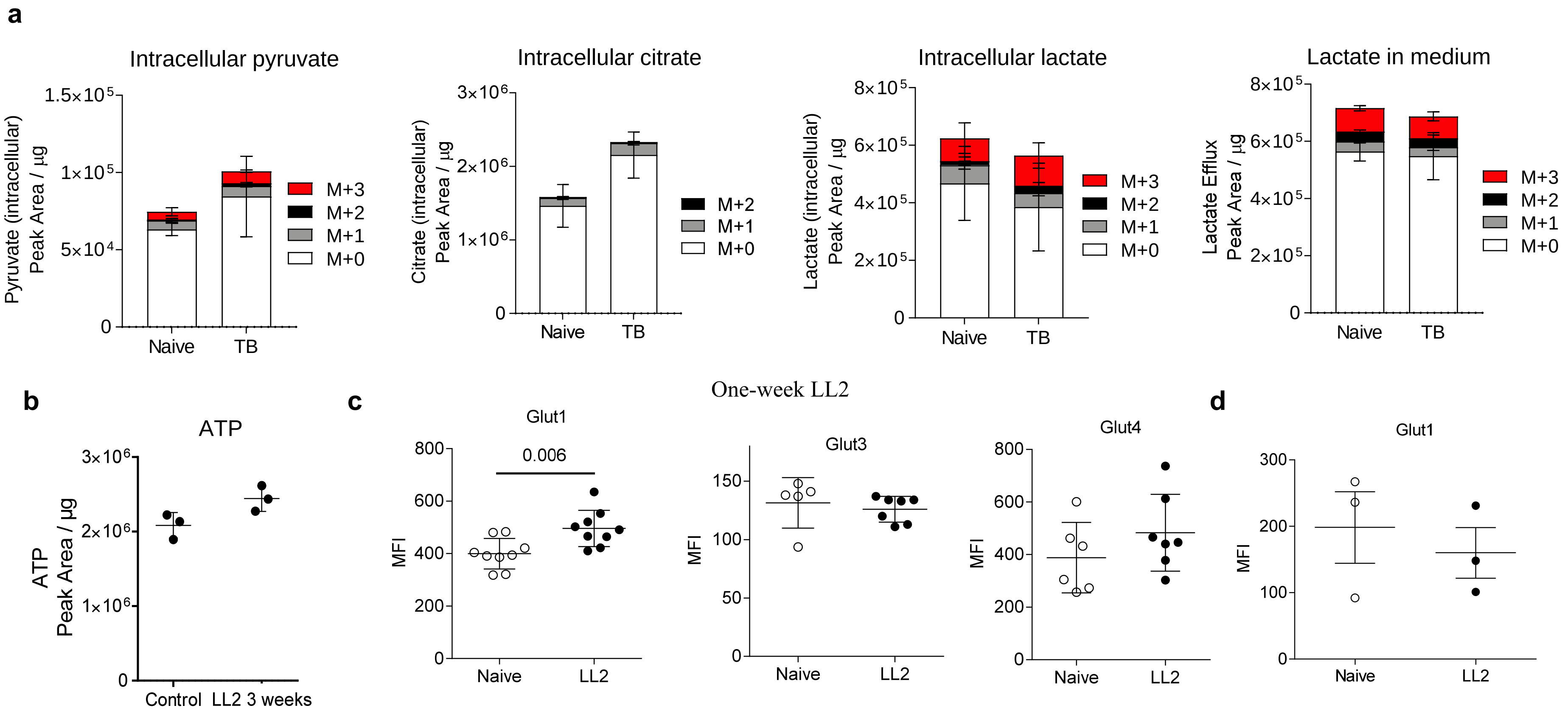

Supplemental Figure 6. Glucose metabolism in neutrophils. a. Flow of $^{13}C_6$-glucose through glycolysis in BM neutrophils from control and TB mice with 3-week s.c. LL2 tumor (n=3). **b.** ATP level in neutrophils (n=3) **c.** Expression of glucose transporter on the surface of neutrophils from naïve and one-week LL2 TB mice (n=7). **d.** Expression of Glut1 on the surface of neutrophils from naïve and three-week LL2 TB mice (n=3). Individual results for each mouse, mean and SD are shown. Significant p values in two-sided Student's t-test are shown.

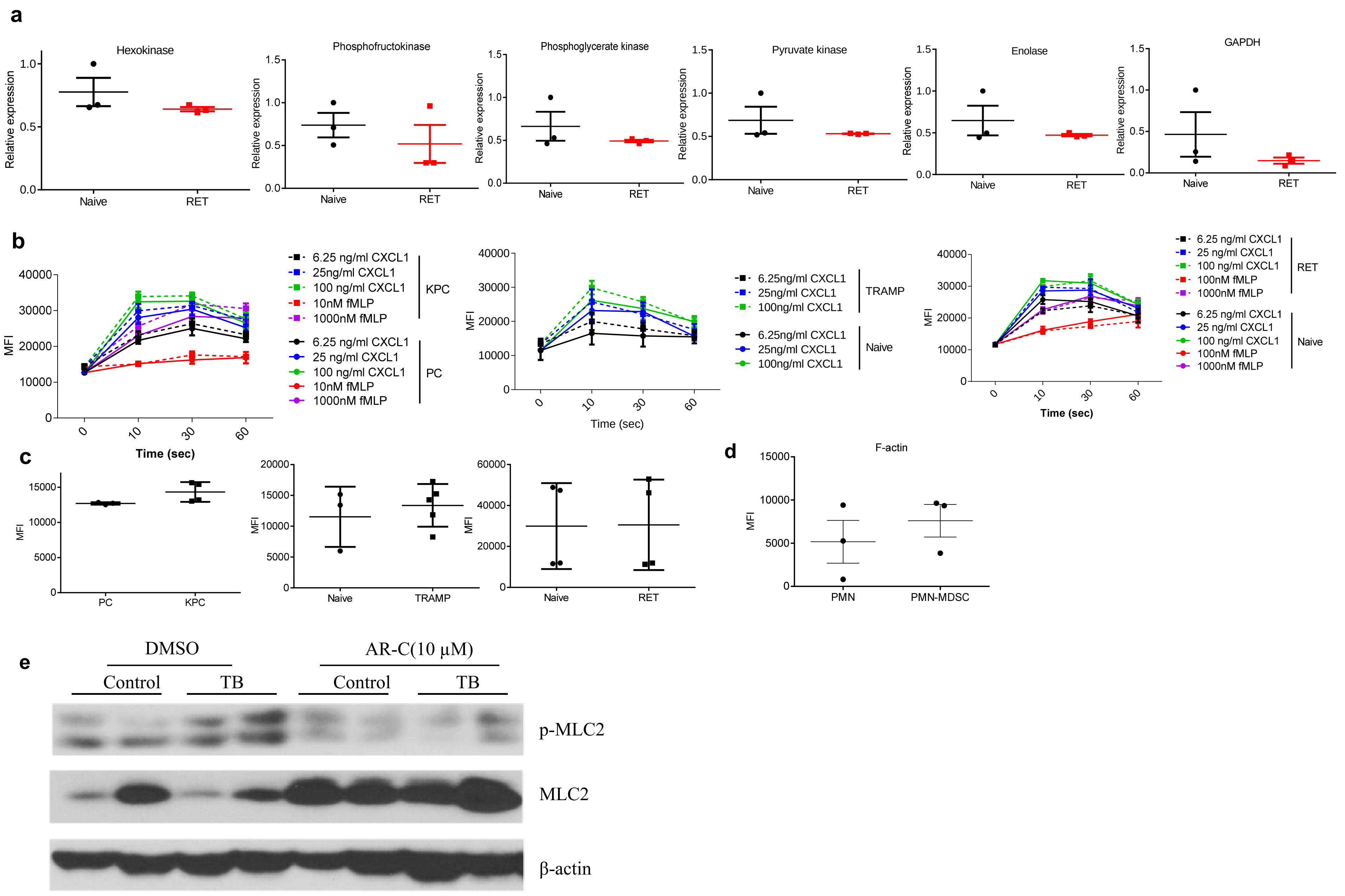

a
Hexokinase
Phosphofructokinase
Phosphoglycerate kinase
Pyruvate kinase
Enolase
GAPDH
Relative expression
Naive
RET
b
6.25 ng/ml CXCL1
25ng/ml CXCL1
100 ng/ml CXCL1
10nM fMLP
1000nM fMLP
KPC
PC
TRAMP
Naive
RET
100nM fMLP
MFI
Time (sec)
c
PC
KPC
TRAMP
d
F-actin
PMN
PMN-MDSC
e
DMSO
AR-C(10 μM)
Control
TB
p-MLC2
MLC2
β-actin

Supplemental Figure 7. Expression of F-actin and genes associated with the glycolytic pathway. a. Expression of indicated genes in neutrophils from control and RET melanoma mice measured in real-time quantitative PCR (n=3). Individual results for each mouse, mean and SD are shown. **b.** F-actin was measured using flow cytometry before and after stimulation with CXCL1 or fMLP in BM neutrophils from indicated tumor models. Typical example of 4 experiments is shown. **c.** F-actin in unstimulated cells evaluated by flow cytometry. Individual results for each mouse, mean and SD are shown. **d.** F-actin in in neutrophils and PMN-MDSC measured by flow cytometry (n=4). Individual results for each mouse, mean and SD are shown. Significant p values in two-sided Student's t-test are shown. **e.** pMLC2 in neutrophils from control tumor-free and one-week LLC TB mice. Neutrophils were treated with vehicle alone or with P2Y2 inhibitor (ARC) for 1 hour before Western blot analysis. Two experiments with similar results were performed.

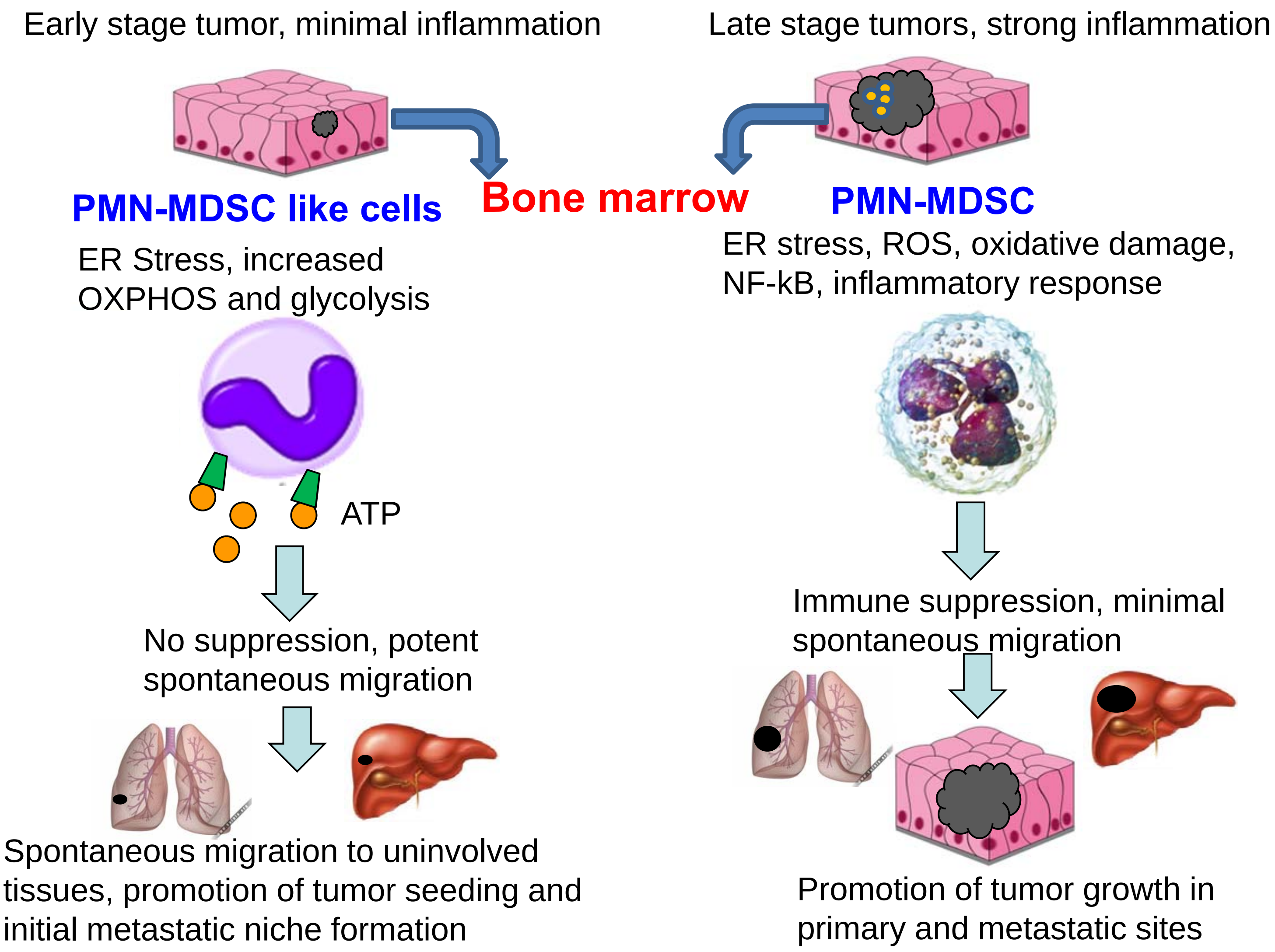

Supplemental Figure 8. Model of PM-LC involvement in tumor progression. During early stages of tumor development, BM neutrophils are characterized by ER stress, and increased OXPHOS and glycolysis, This results in increased spontaneous motility mediated by ATP and purinergic receptors in autocrine and paracrine fashion. These cells actively migrate to uninvolved tissues and promoted tumor cell seeding and formation of metastasis. At later stages, when tumor burden is bigger and tumors are infiltrated with immune cells or contain necrotic loci with associated inflammation, it results in generation of PMN-MDSC, characterized by immune suppressive activity, activation of pro-inflammatory and oxidative damage signaling. These cells actively migrate to the sites with established tumors (primary of metastatic) and promote further tumor growth.

Migration - Naive and RET Combined.mp4

Supplemental Video. Time lapse video demonstrating spontaneous movement of neutrophils from naïve and RET TB mice. Scale bar = 50 µm

Supplemental Table 1. List of reagents

Company	Antibody name	Catalog number	Clone
BD Biosciences	CD45.1-FITC	553775	A20
	CD45.2-APC-Cy7	560694	104
	Ly-6G-PE	551461	1A8
	CD14-APC-Cy7	557831	MϕP-9
	Ly6G-APC	560599	1A8
	Mouse IgG1, κ isotype control-FITC	555909	MOPC-21
	CXCR2-FITC	551126	6C6
	Cytofix/Cytoperm Solution	554722	
	Perm/Wash Buffer	554723	
BioLegend	CD15-PerCP-Cy5.5	323020	SSEA-1
	CXCR1-PE	320608	None
	Mouse IgG2b, κ isotype control-PE	400314	MPC-11
	CXCR4-PE	306505	None
	Mouse IgG2a, κ isotype control-PE	400214	MOPC-173
	CD11b-BV421	101236	M1/70
R&D Systems	CXCR2-PE	FAB2164P	242216
	CXCR1-PE	FAB8628P	1122A
ThermoFisher Scientific	Phalloidin-AF488	A12379	
	Aqua Live / Dead Fixable 405	L34966	None
	DAPI	D3571	None
Novus Biologicals	MitoTracker^{TM} Green FM	M7514	None
	Glut1	NB110-39113	None
	Glut4	NBP1-49533	None
Abcam	Glut3-FITC	ab136180	None
Sigma	Adenosine 5′-diphosphate sodium salt	A2744	None

Supplemental Table 2. Primers sequence

**Glut1**	**Fwd: AGCCCTGCTACAGTGTAT**
	Rev: AGGTCTCGGGTCACATC
**Glut3**	Fwd: ATGGGGACAACGAAGGTGAC
	Rev: CAGGTGCATTGATGACTCCAG
**HIF1α**	Fwd: TCTCGGCGAAGCAAAGAGTC
	Rev: AGCCATCTAGGGCTTTCAGATAA
**Hexokinase**	Fwd: ATGATCGCCTGCTTATTCACG
	Rev: CGCCTAGAAATCTCCAGAAGGG
**Phosphofructokinase**	Fwd: CATCGCCGTGTTGACCTCT
	Rev: CCCGTGAAGATACCAACTCGG
**GAPDH**	Fwd: CCCTTAAGAGGGATGCTGCC
	Rev: ACTGTGCCGTTGAATTTGCC
**Phosphoglycerate kinase**	Fwd: CCCAGAAGTCGAGAATGCCTG
	Rev: CTCGGTGTGCAGTCCCAAA
**Enolase**	Fwd: AGTACGGGAAGGACGCCACCA
	Rev: GCGGCCACATCCATGCCGAT
**Pyruvate kinase**	Fwd: GCCGCCTGGACATTGACTC
	Rev: CCATGAGAGAAATTCAGCCGAG
**β-actin**	Fwd: CCTTCTTGGGTATGGAATCCTGT
	Rev: GGCATAGAGGTCTTTACGGATGT
